# Soil Health Management Enhances Microbial Nitrogen Cycling Capacity and Activity

**DOI:** 10.1128/mSphere.01237-20

**Published:** 2021-01-13

**Authors:** Jialin Hu, Virginia L. Jin, Julie Y. M. Konkel, Sean M. Schaeffer, Liesel G. Schneider, Jennifer M. DeBruyn

**Affiliations:** a Department of Biosystems Engineering & Soil Science, University of Tennessee, Knoxville, Tennessee, USA; b USDA—Agricultural Research Service, Agroecosystem Management Research Unit, University of Nebraska-Lincoln, Lincoln, Nebraska, USA; c Department of Animal Science, University of Tennessee, Knoxville, Tennessee, USA; University of Wisconsin-Madison

**Keywords:** soil health, conservation agriculture, soil nitrogen cycle, soil microbes, quantitative PCR (qPCR), quantitative reverse transcription PCR (qRT-PCR), agroecosystems, denitrification, nitrification, nitrogen fixation, soil microbiology

## Abstract

Soil microbial transformations of nitrogen (N) can be affected by soil health management practices. Here, we report *in situ* seasonal dynamics of the population size (gene copy abundances) and functional activity (transcript copy abundances) of five bacterial genes involved in soil N cycling (ammonia-oxidizing bacteria [AOB] *amoA*, *nifH*, *nirK*, *nirS*, and *nosZ*) in a long-term continuous cotton production system under different management practices (cover crops, tillage, and inorganic N fertilization). Hairy vetch (Vicia villosa Roth), a leguminous cover crop, most effectively promoted the expression of N cycle genes, which persisted after cover crop termination throughout the growing season. Moreover, we observed similarly high or even higher N cycle gene transcript abundances under vetch with no fertilizer as no cover crop with N fertilization throughout the cover crop peak and cotton growing seasons (April, May, and October). Further, both the gene and transcript abundances of *amoA* and *nosZ* were positively correlated to soil nitrous oxide (N_2_O) emissions. We also found that the abundances of *amoA* genes and transcripts both positively correlated to field and incubated net nitrification rates. Together, our results revealed relationships between microbial functional capacity and activity and *in situ* soil N transformations under different agricultural seasons and soil management practices.

**IMPORTANCE** Conservation agriculture practices that promote soil health have distinct and lasting effects on microbial populations involved with soil nitrogen (N) cycling. In particular, using a leguminous winter cover crop (hairy vetch) promoted the expression of key functional genes involved in soil N cycling, equaling or exceeding the effects of inorganic N fertilizer. Hairy vetch also left a legacy on soil nutrient capacity by promoting the continued activity of N cycling microbes after cover crop termination and into the main growing season. By examining both genes and transcripts involved in soil N cycling, we showed different responses of functional capacity (i.e., gene abundances) and functional activity (i.e., transcript abundances) to agricultural seasons and management practices, adding to our understanding of the effects of soil health management practices on microbial ecology.

## Abstract (precis)

Conservation agriculture practices that promote soil health have distinct and lasting effects on microbial populations involved with soil nitrogen (N) cycling. In particular, using a leguminous winter cover crop (hairy vetch) promoted the expression of key functional genes involved in soil N cycling, equaling or exceeding the effects of inorganic N fertilizer.

## INTRODUCTION

Nitrogen (N) is a major nutrient for plant growth, and its transformation and availability in soil is a key factor in terrestrial ecosystem productivity (1). Nitrogen fertilizer has been widely used to improve crop yields in agroecosystems, but losses of excess N result in the eutrophication of waterbodies and exacerbate atmospheric concentrations of the greenhouse gas nitrous oxide (N_2_O) (2). Conservation agriculture and soil health practices, such as growing cover crops, reducing N fertilization, and minimizing tillage, ensure mineral nutrients can be properly used by crops and do not become surplus or deficient (3).

The C-to-N ratios of plant residues can drive the net balance of soil mineral N by affecting microbial mineralization and immobilization of N (4). Cover crops, in addition to the main crop, can take up soil nitrate (NO_3_^−^) to reduce N losses via leaching and denitrification (5). Further, leguminous cover crops can supplement crop N requirements through biological N fixation, thereby reducing exogenous N fertilizer requirements and associated N losses (6). The effects of tillage intensity on N losses through N_2_O emissions are cropping system specific, but reduced or no tillage generally mitigates N_2_O emissions compared to conventional tillage, especially in long-term (>10 years) production systems (7, 8).

Conservation agriculture practices can change soil properties, which are important factors influencing microbial functional groups involved in soil N cycling (9). For example, increased soil organic matter (SOM) and soil water content (SWC) under reduced tillage and winter cover cropping can create nutrient-rich anaerobic microsites that favor microbial denitrification (10, 11). N limitation caused by reduced N fertilization may decrease the population size of ammonia-oxidizing bacteria (AOB) (12) but promote the activity of diazotrophs for N fixation (13).

The abundance and expression of genes encoding key enzymes involved in soil N transformations have been widely used to evaluate the abundances and activity of microbial populations involved in specific N cycling processes (14). For example, different microbial assemblages are responsible for N fixation, nitrification, and denitrification (15). Biological N fixation is the reduction of atmospheric N_2_ to biologically available ammonium. Molecular analysis of N-fixing bacteria often targets *nifH* genes encoding a subunit of nitrogenase reductase, which catalyzes the reduction of dinitrogen to ammonia (16–18). Nitrification is the process of converting ammonia to nitrate and is one of the major processes contributing to N_2_O production (19–22) and nitrate leaching (23) in soils. The first and rate-limiting step of nitrification is microbial oxidation of ammonia to hydroxylamine catalyzed by ammonia monooxygenases (AMO). Thus, molecular analysis of ammonia-oxidizing bacteria (AOB) and archaea (AOA) commonly targets *amoA* genes encoding subunit A of AMO (24, 25). Although AOA may be more numerically abundant than AOB in soil (26), AOB have been shown to be generally more responsive than AOA to soil management practices, such as N fertilization (27–29) and tillage (30), and were shown to be functionally dominant for ammonia oxidation in agricultural topsoils (31). Denitrification is the stepwise reduction of nitrate to nitrite, nitric oxide, nitrous oxide, and dinitrogen gas under anaerobic conditions and is catalyzed by nitrate, nitrite, nitric oxide, and nitrous oxide reductases, respectively (32). Reduction of nitrite to nitric oxide is catalyzed by two structurally different nitrite reductases (NIR) carried by different taxa as follows: (i) Cu-containing NIR (CuNIR) encoded by a single gene *nirK*, sometimes accompanied by gene *nirV*, and (ii) cytochrome *cd*_1_ NIR (cdNIR) encoded by at least 10 genes (*nirSECFDLGHJN*) with *nirS* gene encoding the functional subunits of cdNIR (33). The reduction of N_2_O to N_2_ gas is catalyzed by nitrous oxide reductase (NOS), which is frequently studied by using the marker gene *nosZ* that encodes the multi-Cu NOS catalytic subunit (34, 35). The variation and the ratio between *amoA*, *nirK*, and *nirS* that are involved in N_2_O production and *nosZ* that is involved in N_2_O reduction have been used to predict system-level N_2_O emissions (36, 37).

The reported effects of conservation soil management practices on important functional groups involved in N cycling are inconsistent. For example, many studies indicate that reduced or no tillage can increase the abundance of both AOA and AOB *amoA* (38), *nirK* (39), *nosZ* (39, 40), and (*nirK* + *nirS*)/*nosZ* ratios (41). Other studies, however, reported a positive effect of no tillage on AOB *amoA* (30) but did not find significant effects of tillage on the abundances of AOA *amoA* (30), *nirK* (42), or *nirS* genes (39). Cover cropping may also affect some populations: *nirK* gene abundances were higher under cereal rye compared to hairy vetch while *nirS* gene abundances were unaffected by cover crop species (43). Nitrogen input can suppress *nifH* abundance when the available N is excessive (44, 45), but it can also improve abundances when available N is limited (46). These inconsistent and sometime contradictory results may be due to a lack of temporal resolution. For example, field trials may not have been conducted long enough to invoke a significant change in the soil system. Moreover, there is seasonal variability in microbial functional dynamics driving N transformations that are not captured if only a single time point sample is taken. Another critical gap in our current understanding of functional group dynamics is the relationship between functional capacity and activity. Many studies have only focused on N cycling functional groups at either the genetic level (i.e., using extracted DNA), which misses changes in activity or expression, or at the transcriptional level (i.e., using extracted RNA), which misses changes in population abundances.

The objectives of our study were to (i) determine the effects of tillage, cover cropping, and N fertilization on abundance and activity of N cycling microbes; (ii) investigate the seasonal dynamics of abundance and activity of N cycling functional groups under long-term conservation soil management; and (iii) identify relationships among the N cycle functional gene and transcript abundances, microbial-controlled N transformation rates, and soil physicochemical parameters. The study was conducted at a long-term (36 years) continuous cotton experiment with different tillage, cover cropping, and N fertilization regimes. To examine the dynamics of soil N cycling functional groups, we used quantitative PCR (qPCR) and real-time quantitative reverse transcription-PCR (qRT-PCR) to target the N cycle genes and transcripts of *nifH*, AOB *amoA*, *nirK*, *nirS*, and *nosZ*. We then explored the seasonal dynamics of soil N cycling populations under these different conservation agricultural management practices and their relationships to soil N pools and fluxes.

## RESULTS

### Soil properties and ambient temperatures.

All of the soil physicochemical parameters and N transformation rates measured in this study showed seasonal dynamics, and most of them were significantly affected by hairy vetch (see Table S2 and S3 in the supplemental material). In particular, increased nitrification and N mineralization rates in vetch plots were mainly observed in cover crop peak season (April) and spring crop transition (May) (Table S3). N fertilization significantly increased the concentration of NO_3_^−^-N, total soil C (TC), total soil N (TN), N_2_O-N, and the rate of incubated nitrification and N mineralization (Table S3). Compared to conventional tillage (CT), no tillage (NT) significantly increased the concentration of total extractable C (TEC), TC, and TN, which was particularly noticeable in April and October for TEC and in May and October for TC and TN (Table S3). Ambient temperatures during the cotton growing seasons (May and October, 16.1 and 17.2°C, respectively) were higher than during the cover crop growing seasons (November and April, 8.3 and 9.4°C, respectively).

### N cycle genes and transcripts.

The abundances of N cycle genes and transcripts were influenced differently by agricultural season and soil management practices. In general, for both relative (normalized to 16S rRNA genes and 16S rRNA) and absolute abundances (per gram dry weight soil), genes were mostly affected by only main effects or two-way interaction effects of agricultural season and soil management practices, while transcripts were affected by much more complex interaction effects among season and management practices (Table 1; see also Table S4 and S5 in the supplemental material).

**TABLE 1:** Results of mixed model ANOVA testing effects of agricultural season and soil management practices on the normalized and absolute abundances of N cycle genes and transcripts^*a*^

Factor	Abundance^*b*^	*nifH*		AOB *amoA*		*nirK*		*nirS*		*nosZ*		16S	
		Gene	Transcript	Gene	Transcript	Gene	Transcript	Gene	Transcript	Gene	Transcript	Gene	Transcript
Season	N	10.97***	71.34***	56.80***	7.91***	12.81***	49.64***	16.04***	62.88***	41.16***	24.29***		
	A	13.44***	62.28***	57.66***	15.97***	12.54***	30.94***	25.48***	64.89***	31.54***	33.34***	9.30***	15.65***
Tillage	N	9.18**	7.12**	0.04	0.02	0.57	0.03	6.71*	0.78	0.51	9.87**		
	A	49.60***	13.20***	6.63*	2.08	17.28***	2.75	59.71***	4.36*	18.30***	17.20***	10.12**	3.81
Cover	N	2.08	4.18*	88.83***	71.70***	1.09	26.28***	2.68	14.81***	6.29**	28.50***		
	A	2.21	10.97***	87.58***	52.55***	0.86	23.39***	3.71*	18.62***	7.74***	27.22***	0.40	8.55***
Nitrogen	N	0.00	0.03	0.16	0.12	2.63	0.89	18.81***	1.22	17.69***	1.42		
	A	4.10*	0.09	5.65*	0.17	2.69	0.88	4.99*	1.25	2.24	1.27	5.18*	0.05
Season × till	N	1.33	2.91*	3.72*	3.74*	0.99	0.42	2.47	1.11	1.77	3.39*		
	A	1.04	3.07*	2.97*	2.68*	0.86	1.81	1.46	0.96	1.52	3.68*	0.55	1.93
Season × cover	N	1.03	2.17*	1.12	8.41***	0.70	5.12***	0.51	4.81***	0.64	7.56***		
	A	0.94	1.27	1.78	4.59***	1.82	3.37**	1.31	5.53***	2.10	4.93***	0.92	2.37*
Season × N	N	0.73	0.88	1.15	4.82**	2.46	0.67	1.88	0.38	2.06	1.60		
	A	0.10	1.60	0.83	1.74	1.64	2.35	1.07	0.92	0.74	3.87*	1.05	1.50
Till × cover	N	0.43	0.61	2.34	0.15	0.04	0.79	0.34	0.77	0.04	1.89		
	A	0.77	1.29	2.02	1.12	0.11	0.16	0.32	1.05	0.07	0.15	0.07	1.70
Till × N	N	0.55	11.63***	0.76	9.99**	0.59	20.20***	0.03	2.48	0.05	11.71***		
	A	0.02	0.56	0.06	0.43	1.09	3.34	2.31	0.07	1.39	0.29	1.55	5.97*
Cover × N	N	0.20	3.86*	13.66***	16.38***	1.58	1.59	4.37*	6.83**	0.67	7.98***		
	A	0.80	2.56	5.51**	8.78***	4.99**	0.69	16.37***	3.99*	3.38*	3.97*	2.30	0.26
Till × cover × N	N	2.10	0.76	2.52	0.23	0.43	1.08	0.96	2.18	0.94	0.45		
	A	2.03	1.05	1.04	0.93	1.79	1.79	0.13	3.56*	0.06	1.74	0.38	0.84
Season × till × cover	N	0.72	5.26***	1.11	6.16***	0.94	9.88***	1.11	4.82***	0.65	4.75***		
	A	1.10	3.66**	1.78	2.33*	0.99	7.46***	0.28	4.39***	0.29	1.82	1.07	2.05
Season × till × N	N	0.23	0.29	0.29	0.38	0.17	1.43	0.89	1.59	0.98	0.44		
	A	0.61	0.08	0.74	0.32	2.57	0.76	2.98	1.04	2.05	0.10	1.52	0.08
Season × cover × N	N	0.93	2.39*	0.89	6.40***	0.60	3.33**	0.42	1.28	0.64	4.18***		
	A	1.63	2.26*	1.18	2.97**	0.59	2.18*	1.03	1.10	0.76	2.60*	0.18	0.82
Season × till × cover × N	N	0.30	0.58	1.67	1.80	0.48	1.21	0.78	2.46*	0.51	2.26*		
	A	0.29	1.90	1.27	1.84	0.64	1.82	0.95	2.94**	0.59	4.49***	0.47	1.79

a ANOVA based on GLIMMIX procedure in SAS. Significance is shown with bold fonts and asterisks. *F* values are reported. Significance levels are as follows: *, *P* value ≤ 0.05; **, *P* value ≤ 0.01; *** *P* value ≤ 0.001.

b N, normalized; A, absolute.

**FIG 1:**
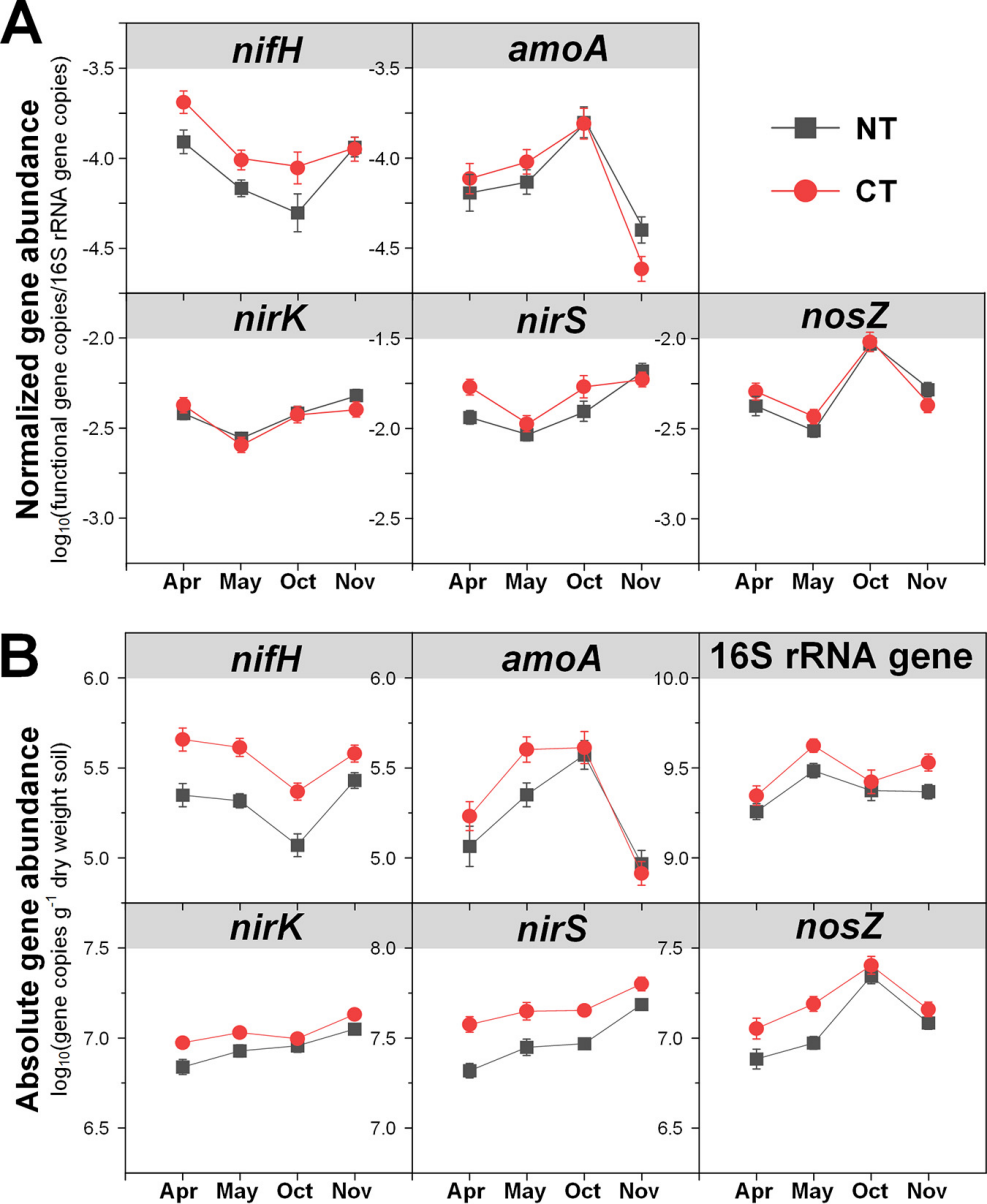
Seasonal dynamics of 16S rRNA gene normalized *nifH*, *amoA*, *nirK*, *nirS*, and *nosZ* gene abundances (A) and absolute abundances of *nifH*, *amoA*, *nirK*, *nirS*, *nosZ*, and 16S rRNA genes (B) in relation to tillage. Points represent the mean ± standard error (*n* = 24). NT, no tillage; CT, conventional tillage.

**FIG 2:**
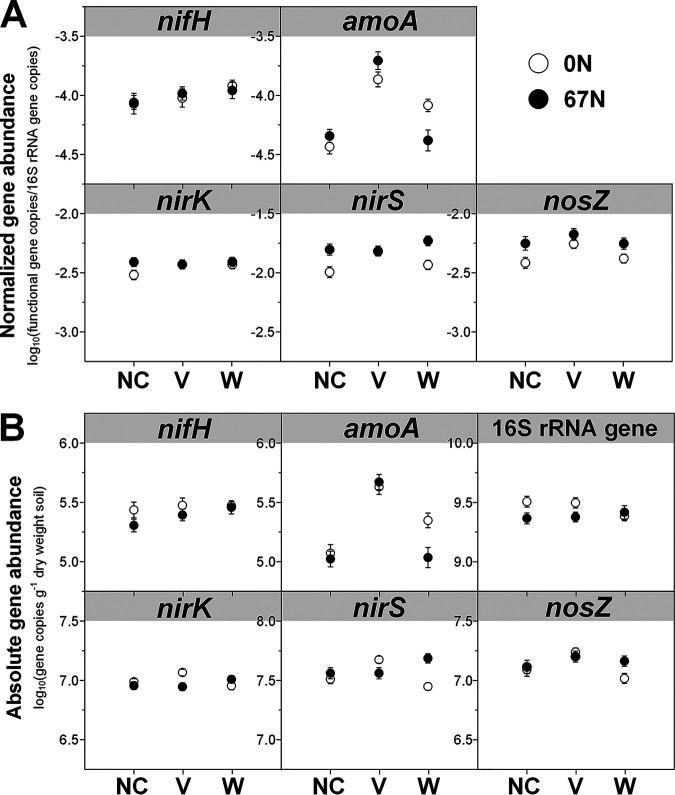
Variation of 16S rRNA gene normalized *nifH*, *amoA*, *nirK*, *nirS*, and *nosZ* gene abundances (A) and absolute abundances of *nifH*, *amoA*, *nirK*, *nirS*, *nosZ*, and 16S rRNA genes (B) in relation to N fertilization rate (0N, no N fertilization; 67N, 67 kg N ha^−1^ fertilization) under different cover crop treatments (NC, no cover; V, vetch; W, wheat). Points represent the mean ± standard error (*n* = 32).

**FIG 3:**
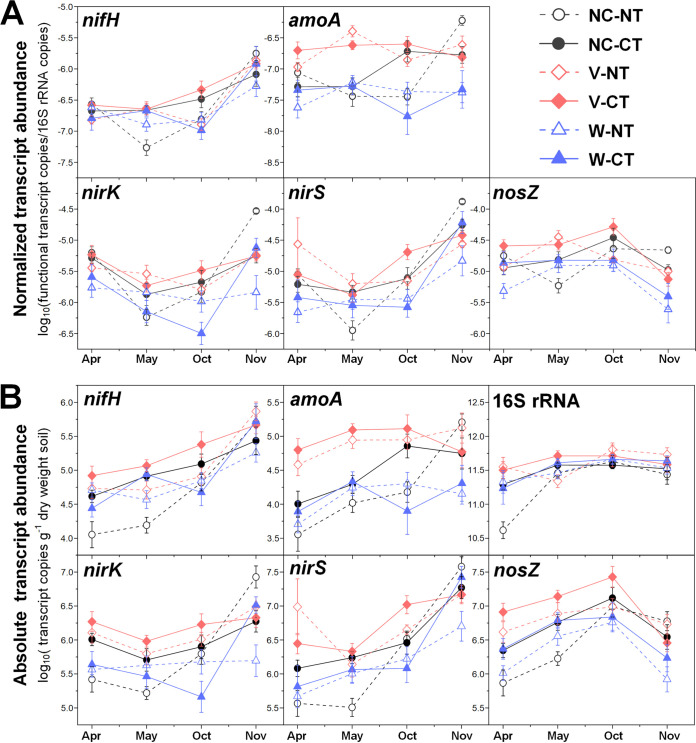
Seasonal dynamics of 16S rRNA normalized transcript abundances of *nifH*, *amoA*, *nirK*, *nirS*, and *nosZ* (A) and absolute transcript abundances of *nifH*, *amoA*, *nirK*, *nirS*, *nosZ*, and 16S rRNA gene (B) in relation to cover crops and tillage. Points represent the mean ± standard error (*n* = 8). NC, no cover; V, vetch; W, wheat; NT, no tillage; CT, conventional tillage.

**FIG 4:**
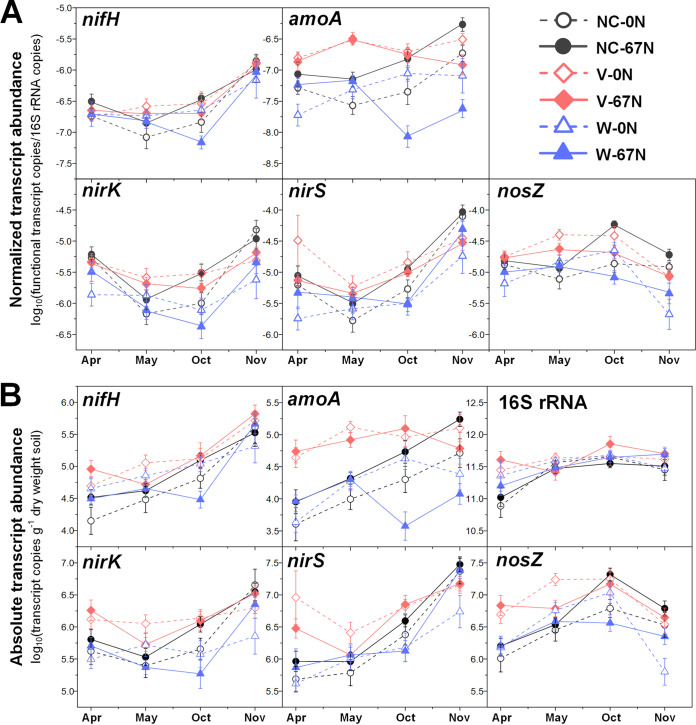
Seasonal dynamics of 16S rRNA normalized transcript abundances of *nifH*, *amoA*, *nirK*, *nirS*, and *nosZ* (A) and absolute transcript abundances of *nifH*, *amoA*, *nirK*, *nirS*, *nosZ*, and 16S rRNA gene (B) in relation to cover crop treatments and N fertilization rate. Points represent the mean ± standard error (*n* = 8). NC, no cover; V, vetch; W, wheat; 0N, no N fertilization; 67N, 67 kg N ha^−1^ fertilization.

Both relative and absolute abundances of marker genes for N fixation (*nifH*) were significantly affected by agricultural season and tillage (Table 1), with highest abundances during cover crop harvest (April) and under CT (Fig. 1). Moreover, although no significant effect of N fertilization on the relative abundance of *nifH* genes was observed (Fig. 2A), absolute abundance of *nifH* was significantly higher under no N fertilization than N fertilization, especially in no cover (NC) and vetch (V) cropping systems (Fig. 2B). Expression of *nifH* had different and more complex dynamics. N fertilization (67 kg N ha^−1^ [67N]) increased *nifH* transcript relative abundance in NT but decreased it in CT (see Fig. S2A in the supplemental material). In general, *nifH* transcript abundances were higher during fall crop transition (November) than other seasons (Fig. 3 and 4). *nifH* transcript abundances were also significantly affected by the interaction of season, cover crop, and tillage (Table 1). The relative abundance of *nifH* transcripts was not significantly different regardless of cover crops or tillage in April but was significantly higher under CT than NT in spring crop transition (May) for NC plots, in cotton peak season (October) for NC and V plots, and in fall crop transition (November) for wheat (W) plots (Fig. 3A). The absolute abundance of *nifH* transcript was significantly higher under CT than NT in April and May for NC plots, in October for V plots, and in November for W plots (Fig. 3B). The interaction of season, cover crop, and N fertilization also significantly affected *nifH* transcripts (Table 1). Fertilizer generally had a positive effect on *nifH* transcripts in NC plots but a negative effect in W plots, especially in October (Fig. 4).

Both relative and absolute abundances of ammonia oxidation gene AOB *amoA* were significantly affected by season combined with tillage (Table 1), with the highest relative abundance in October under both CT and NT and lowest in November under CT (Fig. 1A). The highest absolute *amoA* abundance was in May under CT and in October under both CT and NT and lowest in November under both CT and NT (Fig. 1B). The *amoA* gene abundances were also significantly affected by the combination of cover crop and N fertilization (Table 1): N fertilization increased *amoA* gene relative abundance under V and NC but decreased both relative and absolute abundances under wheat (Fig. 2). Similar to *nifH*, addition of N fertilization increased relative abundance of *amoA* transcripts under NT but decreased under CT (Fig. S2A). Their absolute abundance was not affected by the interaction of N fertilization and tillage (Fig. S2B). In addition, *amoA* transcript abundances were significantly affected by the combination of season, cover crop, and tillage (Table 1). In April and May, *amoA* transcript abundances were highest under V in both NT and CT (Fig. 3). After a lag in April and May, NC plots had increased *amoA* transcripts in October under CT and in November under NT (Fig. 3). The interaction of season, cover crop, and N fertilization also significantly affected *amoA* transcripts (Table 1). Addition of fertilizer significantly increased *amoA* transcript relative abundance under W in April and under NC in May, though not to the same level as V (Fig. 4A). In October and November, 67N continued to exhibit increased relative abundance of *amoA* transcripts under NC; in contrast, 67N caused a decrease under W in October and November and V in November (Fig. 4A). The absolute abundance of *amoA* transcripts showed similar trends to their relative abundance (Fig. 4B).

The abundances of nitrite reduction gene *nirK* varied by agricultural season (Table 1), with lowest relative and absolute abundances in May and April, respectively (Fig. 1). CT increased absolute abundance rather than relative abundance of *nirK* genes (Fig. 1). N fertilization significantly increased their absolute abundance under V (Fig. 2B). The relative abundance of *nirK* transcripts increased with fertilization in NT plots but decreased under CT (Fig. S2A). In addition, the *nirK* transcript abundances were significantly affected by the combination of season, cover crop, and tillage (Table 1). In April, May, and October, *nirK* transcript abundances were generally highest under V, which was more obvious for their absolute abundance (Fig. 3). In November, CT plots had similar levels of *nirK* transcripts regardless of cover, while NT plots had much more variable abundances between cover crops (Fig. 3). The interaction of season, cover crop, and N fertilization rate also significantly affected both relative and absolute abundances of *nirK* transcripts (Table 1). Fertilization generally increased *nirK* transcripts under W in April and November but decreased in May and October (Fig. 4).

The abundances of nitrite reduction gene *nirS* were significantly affected by agricultural season and tillage (Table 1), with higher abundances in November and under CT (Fig. 1). *nirS* gene abundances were also significantly affected by the combination of cover crop and N fertilization rate (Table 1): N fertilization increased *nirS* gene relative abundance in NC and W plots but had no effect in V plots (Fig. 2A); N fertilization increased *nirS* gene absolute abundance in W plots but decreased in V plots (Fig. 2B). *nirS* transcript abundances were significantly affected by the interaction between agricultural season and all three soil management practices (Table 1). The highest transcript relative abundance of *nirS* was observed in NT-V-0 kg N ha^−1^ (0N), NT-V-67N, CT-V-0N, and NT-NC-67N in April, May, October, and November, respectively, while the highest absolute abundance was observed in NT-V-0N, CT-V-0N, CT-V-67N, and NT-NC-0N in April, May, October, and November, respectively (Table S4 and S5).

The abundances of nitrous oxide reduction gene *nosZ* were significantly affected by agricultural season (Table 1), with both relative and absolute abundances highest in October (Fig. 1). Their absolute abundance was higher in CT than NT (*P *<* *0.0001) (Fig. 1B). *nosZ* gene relative abundance was highest under vetch (*P *=* *0.0024) and 67N treatments (*P *<* *0.0001), while absolute abundance was only increased by 67N under wheat (Fig. 2). As with *nirS* transcripts, there was a significant interaction between agricultural season and the three soil management practices on *nosZ* transcript abundances (Table 1). *nosZ* transcript relative abundance was highest in NT-NC-67N in April and November and CT-V-0N in May and October and lowest in NT-W-0N in April and November, NT-NC-0N in May, and CT-W-67N in October (Table S4). *nosZ* transcript absolute abundance was highest in CT-V-67N in April and October, CT-V-0N in May, and NT-V-67N in November and lowest in NT-NC-0N in April and May, CT-W-67N in October, and CT-W-0N in November (Table S5).

### 16S rRNA genes and 16S rRNA.

The abundance of 16S rRNA genes showed significant seasonal dynamics (*P *< 0.0001). Highest abundances were observed in May and lowest in April (Table S5). Compared to NT, CT slightly but significantly promoted 16S rRNA gene abundances from a mean of 2.35 × 10^9^ copies g^−1 ^dry weight soil in NT to 3.02 × 10^9^ copies g^−1 ^dry weight soil in CT (Table S5). 16S rRNA gene copy abundances decreased from 2.92 × 10^9^ copies g^−1 ^dry weight soil in 0N to 2.43 × 10^9^ copies g^−1 ^dry weight soil in 67N (Table S5). 16S rRNA copy numbers were significantly affected by the combination of season and cover crop (*P *< 0.05), which was lowest in April under NC (9.01 × 10^10^ copies g^−1 ^dry weight soil) and highest in October in V (5.74 × 10^11^ copies g^−1 ^dry weight soil; see Table S5). Moreover, 67N increased 16S rRNA abundances in CT but decreased them in NT (Fig. S2). Unlike N cycle gene transcripts, the abundance of 16S rRNA was not significantly affected by the interaction of season, tillage, and cover crop/N fertilization (Table 1; Fig. 3 and 4).

### Cover crop effects on N cycling microbial activity.

In general, V plots tended to exhibit the highest N cycle gene transcript abundances across seasons. The positive effect of 67N addition on N cycle gene transcripts was primarily observed in plots with no cover crops (NC), especially during cotton peak season (October) (Fig. 4). It was notable that fertilization did not have a positive effect in V plots, with V-0N exhibiting similarly high or even higher N cycle gene transcript abundances as V-67N, especially during the cotton growing season (May and October) (Fig. 4). We also observed similarly high or even higher N cycle gene transcript abundances under vetch with no fertilizer (V-0N) as no cover crop with N fertilization (NC-67N) throughout the cover crop harvest and cotton growing seasons (April, May, and October) (Fig. 4).

**FIG 5:**
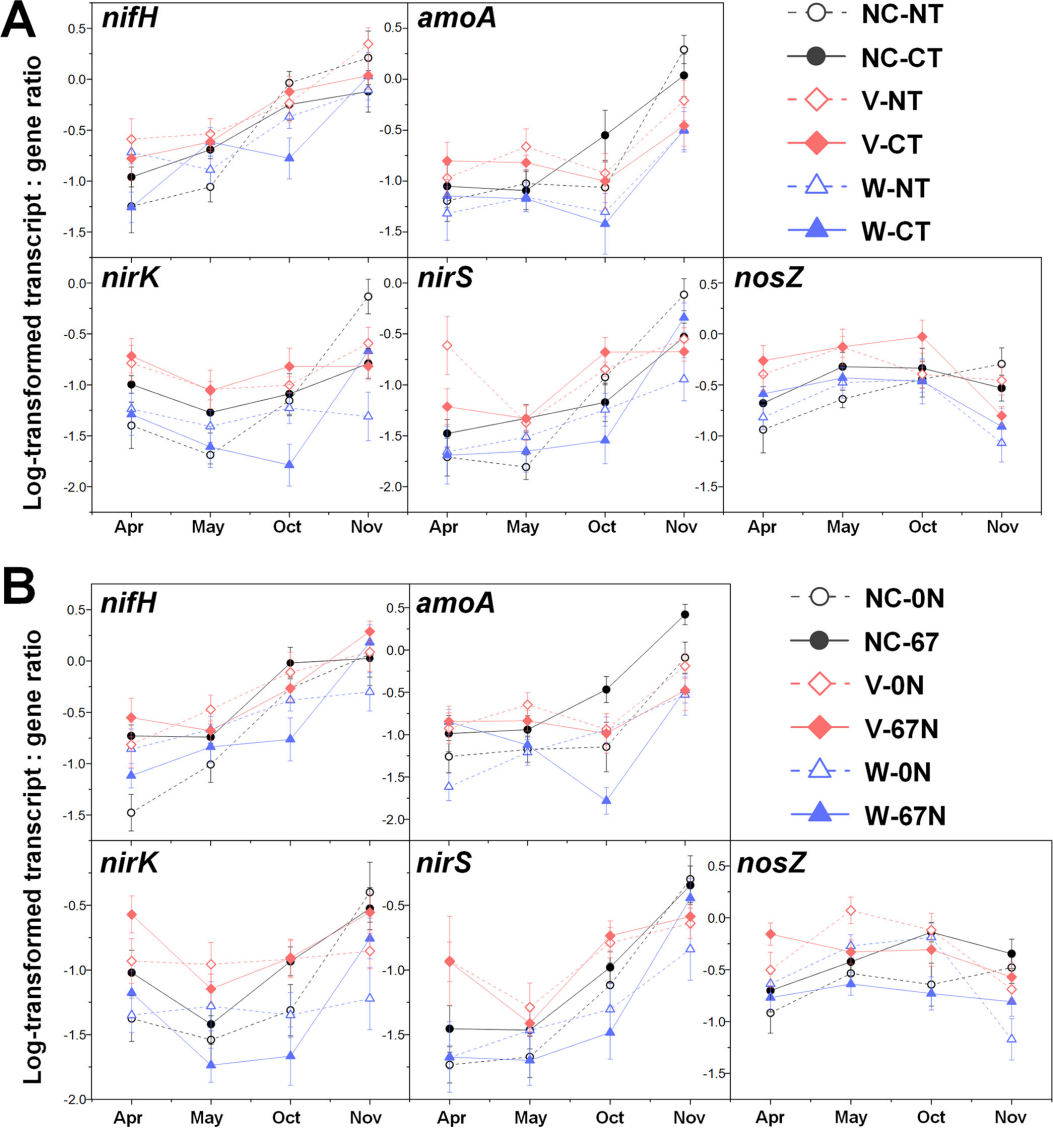
Seasonal dynamics of transcript/gene ratio of *nifH*, *amoA*, *nirK*, *nirS*, and *nosZ* (B) in relation to cover crops and tillage (A) and in relation to cover crops and N fertilization (B). Points represent the mean ± standard error (*n* = 8). NC, no cover; V, vetch; W, wheat; NT, no tillage; CT, conventional tillage.

The transcript/gene ratios of N cycle genes also generally remained higher in V plots under the treatment combination of cover crop with tillage and with N fertilization in April, May, and October (Fig. 5; see Table S6 in the supplemental material), reconfirming the enhancing effect of vetch on N cycle gene expressions. Moreover, the seasonal dynamics of transcript/gene ratio in relation to cover crop with tillage/N fertilization was generally similar to the trends in transcript absolute abundances (Fig. 5), indicating that season and soil management practices had stronger effects at the transcript level compared to the gene abundance level.

### Variability of N cycling microbial functional potential and activity.

**FIG 6:**
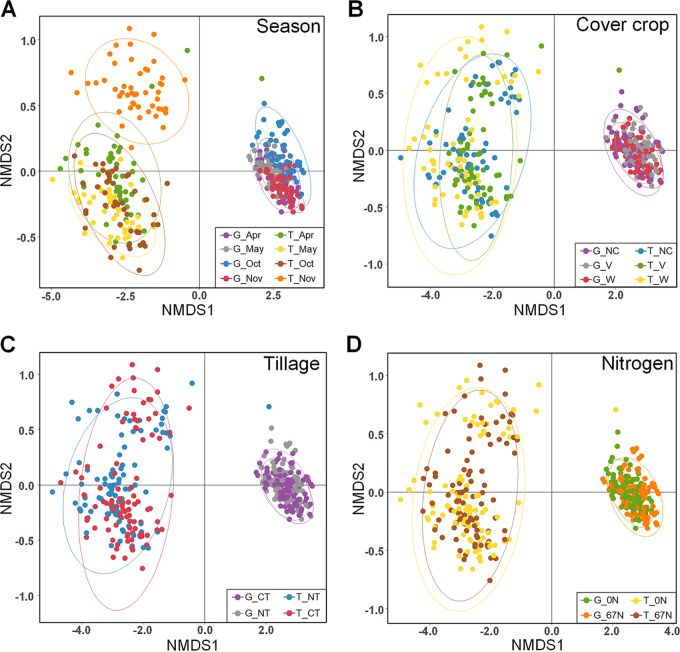
NMDS of Bray-Curtis distances between N cycling communities based on abundances of five N cycling genes (G) and transcripts (T) (stress = 0.015). Points on the ordination plots are colored by season (A), cover crop treatment (B), tillage (C), and fertilization rate (D). NC, no cover; V, vetch; W, wheat; NT, no tillage; CT, conventional tillage; 0N, no N fertilization; 67N, 67 kg N ha^−1^ fertilization. Ellipses represent 95% confidence interval of each group.

Nonmetric multidimensional scaling (NMDS) analysis showed the variability of the nitrogen cycling bacterial populations in terms of functional potential (i.e., based on the five gene relative abundances) and functional activity (i.e., based on the five transcript relative abundances) (Fig. 6). As expected, the statistical dispersion, or variability, of the populations by activity was consistently and significantly higher than for functional potential (dispersion index = 0.3926 for transcripts and 0.2168 for genes; *P* < 0.001). Analysis of similarities (ANOSIM) testing showed overall significant differences in functional potential by season (*R* = 0.1748; *P* = 0.001), tillage (*R* = 0.0222; *P* = 0.020), and N fertilization (*R* = 0.0401; *P* =* *0.003) but not cover crop. The population distribution by activity was significantly affected by season (*R* = 0.2656; *P* = 0.001), cover crop (*R* = 0.0495; *P* = 0.001), and tillage (*R* = 0.0205; *P* = 0.016) but not fertilization (see Table S7 in the supplemental material).

### Relationships of soil N pools and processes and soil properties with functional gene abundance and expression.

**FIG 7:**
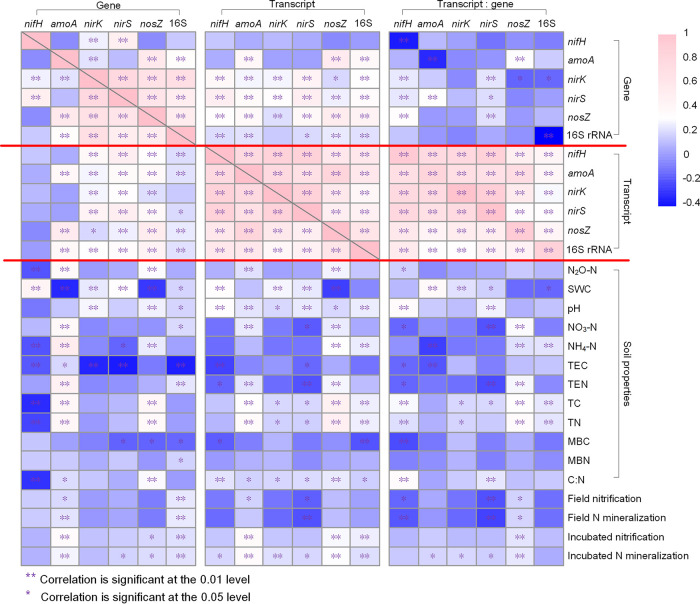
Heatmap showing correlation among genes, gene transcripts, transcript/gene ratios, and soil properties. SWC, soil water content; TC, total carbon; TN, total nitrogen; TEC, total extractable carbon; TEN, total extractable nitrogen; MBC, microbial biomass carbon; MBN, microbial biomass nitrogen.

Correlation heatmaps showed significant correlations among absolute abundances of N cycle genes and their transcripts, transcript/gene ratio, and soil properties (Fig. 7). We observed significant positive correlations among the five gene transcripts and significant positive correlations between N cycle genes and their corresponding transcripts for all gene targets except *nifH* (Fig. 7). Moreover, the transcript/gene ratios of N cycle genes were more related to their transcript abundances than gene abundances, reconfirming that transcript variation depended more on changes in expression rather than population sizes.

N cycle genes and their transcripts, as well as their transcript/gene ratios, showed different patterns of correlations with soil properties (Fig. 7). The gene and transcript abundances of *nifH*, *nirK*, and *nirS* were all positively correlated with SWC (Fig. 7). *amoA* gene and transcript abundances were both significantly and positively correlated with N_2_O-N, NO_3_^−^-N, field nitrification rate, and incubated nitrification rate (Fig. 7). Notably, *amoA* gene was positively correlated with NH_4_^+^-N (*R* = 0.494) while *amoA* transcript had no relationship with NH_4_^+^-N, and the transcript/gene ratio of *amoA* was negatively correlated with NH_4_^+^-N (*R* = −0.260), indicating that although *amoA* gene abundance was promoted by NH_4_^+^, its expression was lower with higher NH_4_^+^-N concentration (Fig. 7). In addition, there were significant positive correlations between N_2_O-N and the gene and transcript abundances of *amoA* and *nosZ* (Fig. 7).

## DISCUSSION

### Seasonal dynamics in abundance and activity of nitrogen cycling populations.

The primary objective of this study was to investigate the impact of soil health management practices on the seasonal population size and activity of soil bacterial communities involved in N transformations by using *nifH*, *amoA*, *nirK*, *nirS*, and *nosZ* as indicators. We found that the N cycling bacterial community activity, as indicated by gene and transcript abundances, respectively, showed seasonal changes.

Without considering soil management practices, the highest functional activity of diazotrophs, as indicated by *nifH* expression, was in November during the fall crop transition, which might be explained by lower N availability at this time. The inhibitory effect of increased N availability (NH_4_^+^-N and/or NO_3_^−^-N) on *nifH* abundance and expression that we observed has been reported in other systems as well (45–48). Diazotrophs will preferentially use easily available N (e.g., nitrate and ammonium), as this takes less energy to assimilate compared to fixing dinitrogen gas (4). Soil freeze-thaw cycles, warming, and N addition can stimulate net N mineralization, increasing N availability (49). Compared to November, the warmer temperatures and N fertilization after cotton planting in May resulted in higher soil N availability during cotton growing seasons, thereby possibly inhibiting the expression of *nifH*. The cotton and winter cover crops take up inorganic and dissolved organic N for growth, leading to increased soil N limitation, especially during the cover crop growing period, which might enhance the competitiveness of diazotrophs and result in their relatively high abundances in April and November. Moreover, N fixation can be affected by soil oxygen content because nitrogenase is an extremely oxygen-sensitive enzyme (4). The high soil water content (SWC) in November may have resulted in lower soil oxygen, contributing to a higher functional activity of diazotrophs. The positive correlation between SWC and *nifH* abundance in our study was also observed in other studies (50, 51). In addition, the relatively high soil C:N ratio and the decomposition of a large amount of plant residues and roots after cotton harvest season (October) may have led to soil N limitation, stimulating the expression of N fixation genes.

The *amoA* gene relative and absolute abundances were both highest in October and lowest in November, which corresponded to the highest soil NH_4_^+^-N concentrations in October and lowest in November. Moreover, a significantly positive correlation between *amoA* gene abundances and NH_4_^+^-N concentrations was observed in our study, indicating that concentrations of NH_4_^+^-N, the substrate for nitrification, influence the abundance of nitrifiers (28, 35). The decrease in *amoA* gene abundance in November may be due to higher SWC and lower soil oxygen availability because AOB are aerobic bacteria (35). The seasonal dynamics of *amoA* transcript relative and absolute abundances both depended on the interaction of cover crops with either tillage or N fertilization rate. However, contrary to *amoA* gene abundance, relative *amoA* expression (normalized to gene abundance) was higher in November than in October and was negatively correlated to NH_4_^+^-N concentrations, indicating that while population sizes of AOB were down, their functional activity was increased. One hypothesis to explain this unexpected result was that ammonia oxidizers increased expression of their functional genes under oxygen and/or nutrient stress. We observed a significant positive correlation between expression level of *amoA* and SWC, which was also reported in other studies. For example, Theodorakopoulos et al. found that >70% water-filled pore space favored transcription of the AOB *amoA* gene (37). A pure culture experiment by Yu and Chandran found that a low level of dissolved oxygen can promote *amoA* transcription in AOB Nitrosomonas europaea (52). In addition, Bollmann et al. reported that during short-term (2 weeks) starvation, the *amoA* mRNA of Nitrosospira briensis remained present, and the ammonia-oxidation functionality was maintained at a high level (53).

The abundances of *nirS* genes and transcripts were greater than those of *nirK* in all agricultural seasons, which suggested that *nirS*-harboring denitrifiers were more predominant at our field site. Both relative and absolute transcript abundances and expressions of *nirK*- and *nirS*-harboring denitrifiers were highest in November, which is likely due to low soil oxygen level resulting from high SWC at that time. The significantly positive correlations of SWC with the transcript abundances and expression levels of *nirK* and *nirS* also support this explanation. Both relative and absolute abundances of *nosZ* genes and transcripts were highest in October. However, the N_2_O-N emissions were highest in both May and October. The not fully consistent seasonal trend between *nosZ* and N_2_O-N emission suggests that the typical *nosZ* (clade I *nosZ*) that we targeted in this study may not independently predict the seasonal dynamics of N_2_O emission, and atypical *nosZ* (clade II *nosZ*) may be playing an important role (54, 55). In fact, several studies have reported that atypical *nosZ* is more abundant than clade I *nosZ* in agricultural soils (56–58).

### Effects of cover crops on abundance and activity of nitrogen cycling populations.

Different responses were observed between the two types of cover crops. In general, compared to winter wheat, hairy vetch had significantly higher transcript abundance and transcript/gene ratios for all N cycle genes. The elevated activity of N cycling microbes under vetch was not limited to just the cover crop period (April) but persisted past cover crop termination through the cotton growing season (October). This supports the idea that leguminous cover crops have a lasting stimulation of overall N cycling microbes in agricultural soil. This is consistent with our previous findings from this site, where we documented significantly increased microbial biomass (both total and microbial biomass N) and enzyme activities in the vetch treatments (59). It may be that the biological N fixation rate stimulated by legumes and the low C:N ratio of legume residues supplied more N to the soil, providing a competitive advantage for N cycling populations (6, 60). Winter wheat is a nonleguminous cover crop with high C:N residues that takes up additional N from soil for growth, which may decrease soil N availability and inhibit the functional gene expression of N cycling microbes (except *nifH*). Some studies observed that leguminous cover crops can promote the release of mineral N and increase denitrification and N_2_O emissions relative to other cover crops or no cover (43, 61, 62). The relatively high and generally similar level of N cycle gene expression under combinations of hairy vetch with no fertilization and no cover crop with N fertilization supports the idea that, in some cases, N-fixing cover crops can replace N fertilization to maintain the N cycling rate.

### Effects of tillage and fertilization on abundance and activity of nitrogen cycling populations.

Overall, tillage only affected the relative abundances of genes *nifH* and *nirS*, but it affected the absolute abundances of all five functional genes and 16S rRNA genes, with higher abundances under conventional tillage than under no tillage. This may be because no-till treatment can decrease surface soil bulk density, increase soil porosity, and result in higher oxygen content in surface soils (63). The aerobic conditions resulting from conventional tillage may suppress the growth of anaerobic N cycling bacteria, such as N fixers and denitrifiers. Another possible explanation is that conventional tillage makes substrates more available for soil microbes by destroying soil aggregates (64, 65), increasing soil microbial populations, including the N cycling populations.

Compared to no fertilization, N fertilization with 67 kg N ha^−1^ only significantly increased the relative abundance of genes *nirS* and *nosZ* but only when no cover crops and wheat were used. This effect was observed only in wheat plots when absolute abundance was taken into consideration. This is partly in accordance with a meta-analysis by Ouyang et al., which showed that N fertilization had no effect on *nifH* abundance, but significantly increased the abundance of *amoA*, *nirK*, *nirS*, and *nosZ* genes, respectively (66). Although some studies using NH_4_NO_3_ as N fertilizer also reported that N fertilization can increase AOB diversity, abundance, and activity (30, 67, 68), we did not observe this in our study, likely because similar concentrations of NH_4_^+^-N and field nitrification rates were observed under both fertilizer treatments. Increased abundance of denitrification genes *nirS* and *nosZ* with N fertilization corresponded to high NO_3_^−^-N concentration and high N_2_O-N emission in fertilized soil.

### Linking the nitrogen cycling groups with nitrogen cycling processes.

In this study, we assessed the relationships between abundance and expression of functional genes and soil properties. We were particularly interested in determining whether N_2_O emissions, a potent greenhouse gas, could be correlated to the population size or activity of bacteria involved in N cycling processes responsible for N_2_O production and transformation. Both the absolute abundances of genes and transcripts of *amoA* and *nosZ* were positively correlated to N_2_O emissions. This suggests that soil AOB populations might be a main contributor to N_2_O production through nitrification, nitrifier denitrification, or both (69, 70). Likewise, Soares et al. also observed that AOB, rather than denitrifiers, were the main contributors to N_2_O emissions in tropical agricultural soils (71). Moreover, research by Theodorakopoulos et al. suggested that AOB *amoA* transcript abundances, rather than gene abundances, were a good indicator of N_2_O emissions, and ammonia oxidation was a major process contributing to N_2_O emissions (37). However, some studies have found no correlations between N_2_O emissions and abundance of related N cycle genes (AOB *amoA*, *nirK*, *nirS*, and *nosZ*) (36, 42). Therefore, the contribution of AOB and denitrifiers to N_2_O emissions might be site-specific and/or influenced by local soil properties and environmental factors. In addition, although we found significant positive correlation between field and incubated net nitrification rates with absolute abundances of both *amoA* genes and transcripts, a disconnection between AOB *amoA* gene abundances and nitrification rates has been observed in some studies (38, 67). This inconsistent result may be site-specific and might be influenced by the proportion of dormant microbes in soils, substrate limitation, microsite heterogeneity, and/or the fact that measured process rates reflect net, rather than gross, rates.

### Conclusions.

In summary, our results showed that the distribution patterns, abundances, and expressions of N cycle functional genes generally changed significantly between seasons and were affected by soil management practices. We demonstrated that leguminous cover crops like hairy vetch can promote functional activity of N cycling populations through the growing season, equaling or exceeding the effects of inorganic N fertilizer. Our findings highlight the fact that both genetic and transcriptional assessments can provide important information about microbial populations and functions. Transcriptional-level study has higher resolution than the genetic level in examining the effects of soil management practices on N cycling communities, with larger variation under different season and management practices observed. In addition, the interaction effects of season and soil management practices on N cycle gene expressions based on relative (normalized by 16S rRNA copies) and absolute (copies per gram soil) transcript abundances showed generally similar trends, indicating that active soil N cycling communities were more responsive than total active soil bacterial populations to agricultural management practices. Together, our work revealed insights into important relationships between microbial functional capacity and activity and associated nitrogen pools and processes in the field, adding to our understanding of the effects of soil health management practices on soil microbial ecology.

## MATERIALS AND METHODS

### Study site, experimental design, and sampling.

This study was conducted at a long-term continuous cotton conservation agriculture field experiment, established in 1981, at the University of Tennessee West Tennessee Research and Education Center (WTREC) in Jackson, Tennessee. Soils at this site are classified as well-drained Lexington silt loams (fine-silty, mixed, thermic, Ultic Hapludalfs) with a 0 to 2% slope. The main crop was rainfed cotton (*Gossypium hirsutum* L.). The field study implemented a randomized complete block design with split-split plot treatment arrangements in four replicates such that N fertilization rate was the main plot treatment, cover crop was the subplot treatment, and tillage was the sub-subplot treatment; a subset of 12 of the 32 treatment combinations was used here (see Fig. S1 in the supplemental material). The 12 cropping system management combinations included 2 types of tillage (conventional tillage [CT], no tillage [NT]); 3 types of winter cover crops (hairy vetch [*Vicia villosa* Roth] [V], winter wheat [Triticum aestivum L.] [W], no cover crop [NC]); and 2 levels of NH_4_NO_3_ fertilization rates (0, 67 kg N ha^−1^ [0N, 67N, respectively]) (Table 2). For tilled plots, tillage was done twice before planting by a standard disc harrow followed by smoothing and breaking up of clods by a triple-K harrow. Field operations for these plots, including cover crop termination, cotton planting, and phosphorus and potassium fertilizer application are described in more detail in previous studies of this site (59, 72).

**TABLE 2:** Summary of 12 cropping system treatments used in this study

Cropping system	Tillage	Winter cover crop	N fertilization
NT-NC-0N	No tillage	No cover	No fertilizer
NT-NC-67N			67 kg N ha^−1^
NT-V-0N		Hairy vetch	No fertilizer
NT-V-67N			67 kg N ha^−1^
NT-W-0N		Winter wheat	No fertilizer
NT-W-67N			67 kg N ha^−1^
CT-NC-0N	Conventional tillage	No cover	No fertilizer
CT-NC-67N			67 kg N ha^−1^
CT-V-0N		Hairy vetch	No fertilizer
CT-V-67N			67 kg N ha^−1^
CT-W-0N		Winter wheat	No fertilizer
CT-W-67N			67 kg N ha^−1^

10.1128/mSphere.01237-20.9 FIG S1 Schematic diagram of the experimental cotton plots located at the University of Tennessee West Tennessee Research and Education Center in Jackson, Tennessee, USA. Download FIG S1, TIF file, 0.2 MB. Copyright © 2021 Hu et al. 2021 Hu et al. This content is distributed under the terms of the Creative Commons Attribution 4.0 International license.

10.1128/mSphere.01237-20.10 FIG S2 16S rRNA normalized transcript abundances of *nifH*, *amoA*, *nirK*, *nirS*, and *nosZ* (A) and absolute transcript abundances of *nifH*, *amoA*, *nirK*, *nirS*, *nosZ*, and 16S rRNA (B) in relation to the combination of tillage and N fertilization (0 or 67 kg N ha^−1^ fertilization). Each value represents the mean ± standard error (*n* = 48). Download FIG S2, TIF file, 0.08 MB. Copyright © 2021 Hu et al. 2021 Hu et al. This content is distributed under the terms of the Creative Commons Attribution 4.0 International license.

Subsamples (2.5-cm diameter by 7.5-cm-long cores) of soil were collected from 10 random locations in each experimental sub-subplot (12 cropping systems × 4 replicates = 48 experimental plots), composited, sieved through a 2-mm mesh, and then refrigerated until analysis. Collections were obtained four times corresponding to different stages of crop growth in 2017, including cover crop harvest (April, immediately following cover crop harvest), spring crop transition (May, after cotton planting and before fertilizer application), cotton crop peak (October, before cotton harvest), and fall crop transition (November, after cotton harvest and before winter cover crop planting). Soil properties measured included N_2_O flux (N_2_O-N), soil water content (SWC), soil pH, NO_3_^−^-N, NH_4_^+^-N, total extractable N (TEN), total extractable C (TEC), total soil C (TC), total soil N (TN), soil ratio of C to N (C:N ratio), microbial biomass N (MBN), microbial biomass C (MBC), field net nitrification rate (field nitrification), field net N mineralization rate (field N mineralization), incubated net nitrification rate (incubated nitrification), and incubated net N mineralization rate (incubated N mineralization). Detailed methods on the measurement of these soil properties are provided in the supplemental material (see Text S1, Methods S1 to S5).

10.1128/mSphere.01237-20.1 TEXT S1 Description of methods used for soil property measurements. Download Text S1, DOCX file, 0.02 MB. Copyright © 2021 Hu et al. 2021 Hu et al. This content is distributed under the terms of the Creative Commons Attribution 4.0 International license.

### Soil DNA and RNA extraction.

Soil genomic DNA was extracted from 0.25 g of soil using the DNeasy PowerSoil Kit (Qiagen, Hilden, Germany) according to the manufacturer’s protocol. DNA concentrations were quantified using NanoDrop One spectrophotometry (NanoDrop Technologies, Wilmington, DE). DNA was stored at −20°C.

Soil RNA was extracted using the RNeasy PowerSoil total RNA kit (Qiagen, Hilden, Germany) according to the manufacturer’s protocol. RNA was eluted in RNase-free water and stored at −80°C. RNA quality was checked by 1% agarose gel electrophoresis and NanoDrop One spectrophotometry (NanoDrop Technologies, Wilmington, DE). Samples were checked for DNA contamination by attempting to amplify the N cycle functional genes by PCR and subsequent agarose gel electrophoresis. Only samples that gave a negative electrophoresis result (i.e., no DNA contamination) were used for reverse transcription. cDNA was produced from RNA using SuperScript IV reverse transcriptase (Invitrogen, Paisley, UK) according to the manufacturer’s protocol. Random hexamer primers (Invitrogen) were used at a final concentration of 2.5 μM per reaction. cDNA samples were diluted to a final concentration of 15 ng μl^−1^ and stored at −20°C.

### Molecular cloning.

Genes *nifH*, *amoA*, *nirK*, *nirS*, and *nosZ* were amplified from extracted soil DNA with previously designed primer pairs and reaction conditions (see Table S1 in the supplemental material). PCR products were purified with QIAquick PCR purification kit (Qiagen), ligated into pCR 4-TOPO vectors (Invitrogen), and transformed into Transform One Shot TOP10 competent cells (Invitrogen) following the manufacturer’s protocol. After shaking at 225 rpm at 37°C for 1 h, cells were spread on LB solid medium containing 50 μg ml^−1^ ampicillin and cultured overnight. Positive clones were grown in liquid LB medium amended with ampicillin medium overnight, and plasmid DNA was extracted using the PureLink quick plasmid miniprep kit (Invitrogen) according to the manufacturer’s instructions. Plasmid DNA concentrations were determined on a Hoefer DQ 200 fluorometer (Hoefer Inc., San Francisco, CA), which uses a Hoechst dye specific for double-stranded DNA that compares DNA fluorescence with a known standard, and then sequenced with primers M13 Forward (−20)/M13 Reverse to confirm that the correct target gene was cloned. The copy numbers of *nifH*, *amoA*, *nirK*, *nirS*, and *nosZ* genes were calculated using the concentration of the extracted plasmid DNA, size (base pairs) of the plasmids containing cloned products, and the molecular weight of DNA. Ten-fold serial dilutions of the plasmid DNA (10^2^ to 10^8^ copies μl^−1^) were used during qPCR assays to generate standard curves for quantifying N cycle genes and transcripts in soil samples.

10.1128/mSphere.01237-20.2 TABLE S1 Primers and PCR conditions used for qPCR and qRT-PCR. Download Table S1, DOCX file, 0.01 MB. Copyright © 2021 Hu et al. 2021 Hu et al. This content is distributed under the terms of the Creative Commons Attribution 4.0 International license.

10.1128/mSphere.01237-20.3 TABLE S2 Results of mixed model ANOVA (based on GLIMMIX procedure in SAS) testing effects of agricultural season and soil management practices on soil parameters. The significances were shown with bold fonts and asterisks. *F* values are reported. Download Table S2, DOCX file, 0.02 MB. Copyright © 2021 Hu et al. 2021 Hu et al. This content is distributed under the terms of the Creative Commons Attribution 4.0 International license.

10.1128/mSphere.01237-20.4 TABLE S3 Mean values of soil parameters in relation to agricultural season and soil management practices. Only treatments or treatment combinations that significantly affected at least one parameter were shown. Different lowercase letters indicate significant differences between treatment levels within groups. The letters were only shown in groups that have significant effects. Means were compared using least significant difference tests (α = 0.05). Download Table S3, DOCX file, 0.04 MB. Copyright © 2021 Hu et al. 2021 Hu et al. This content is distributed under the terms of the Creative Commons Attribution 4.0 International license.

10.1128/mSphere.01237-20.5 TABLE S4 Mean values of log-transformed relative abundances of N cycle genes and their transcripts (normalized by 16S rRNA genes and 16S rRNA) in relation to agricultural season and soil management practices. Download Table S4, DOCX file, 0.05 MB. Copyright © 2021 Hu et al. 2021 Hu et al. This content is distributed under the terms of the Creative Commons Attribution 4.0 International license.

10.1128/mSphere.01237-20.6 TABLE S5 Mean values of log-transformed mean absolute abundances of N cycle genes and their transcripts in relation to agricultural season and soil management practices. Download Table S5, DOCX file, 0.06 MB. Copyright © 2021 Hu et al. 2021 Hu et al. This content is distributed under the terms of the Creative Commons Attribution 4.0 International license.

10.1128/mSphere.01237-20.7 TABLE S6 Mean values of log-transformed transcript/gene ratios of N cycle genes in relation to agricultural season and soil management practices. Download Table S6, DOCX file, 0.03 MB. Copyright © 2021 Hu et al. 2021 Hu et al. This content is distributed under the terms of the Creative Commons Attribution 4.0 International license.

10.1128/mSphere.01237-20.8 TABLE S7 ANOSIM analysis of the distribution patterns of N cycling functional groups in four agricultural seasons and under different soil management practices. Download Table S7, DOCX file, 0.02 MB. Copyright © 2021 Hu et al. 2021 Hu et al. This content is distributed under the terms of the Creative Commons Attribution 4.0 International license.

### Quantitative analysis of N cycle genes and gene transcripts.

For both DNA and cDNA samples, qPCR of *nifH*, *amoA*, *nirK*, *nirS*, and *nosZ* were performed on a CFX96 Optical real-time detection system (Bio-Rad, Laboratories Inc., Hercules, CA, USA). The 20-μl qPCR reaction mixture contained 10 μl Maxima SYBR green qPCR master mix (2×) (Thermo Scientific, USA), 1 μl PCR forward and reverse primer (both 10 μM), 2.5 μl DNA or cDNA template, and 5.5 μl nuclease-free water. Primer sets and reaction parameters used are listed in the supplemental material (see Table S1). Following the reactions, a melt curve analysis was performed to confirm the specificity of the PCR product for each real-time PCR amplification. Quantitative PCR of 16S rRNA genes and 16S rRNA was done using Femto bacterial DNA quantification kit (Zymo Research Corp., CA, USA) following the manufacturer’s protocols.

Both absolute abundances (copies per gram dry weight soil) and relative abundances (normalized to 16S rRNA genes or to 16S rRNA) of functional genes and transcripts were analyzed and compared to ensure the reliability of the result. Absolute abundance helped to achieve better understanding of the variation of N cycling bacteria with seasons and management practices, while relative abundance mitigated the impact of DNA and RNA extraction efficiency on data accuracy and revealed the relative changes of N cycling population within the total soil bacteria. The relative abundance of N cycle genes and transcripts used in this study were calculated as follows.

$$\text{NGA =}\frac{\text{N cycle gene copies/}\mu \text{l DNA extract}}{\text{16S rRNA gene copies/}\mu \text{l DNA extract}}$$

$$\text{NTA = }\frac{\text{N cycle transcript copies/}\mu \text{l RNA extract}}{\text{16S rRNA copies/}\mu \text{l RNA extract}}$$

where NGA is the relative abundance of the gene, and NTA is the relative abundance of the gene transcript.

The transcript-to-gene copy number ratio (transcript/gene ratio) for each gene was also calculated to provide a means to determine whether transcript variation was due to changes in population sizes (i.e., genes) or changes in expression (i.e., transcripts) (73) and was calculated as follows.

$$\text{Transcript/gene ratio =}\frac{\text{transcript copies/g dry weight soil}}{\text{gene copies/g dry weight soil}}$$

### Statistical analysis.

Statistical analyses to test the effects of agricultural season and management practices on the abundance and expression of N cycle genes were performed in SAS 9.4 (SAS Inst., Cary, NC, USA). The relative and absolute abundances of functional genes and transcripts as well as their transcript/gene ratio were both log transformed to achieve normal distributions of residuals. These log-transformed data were analyzed using a repeated-measures mixed model analysis of variance (ANOVA) within the GLIMMIX procedure. The fixed effects included season, tillage system, cover crop, and N fertilizer rate, as well as all interactions. Random effects included the block, block by nitrogen within cover crop, and the block by tillage by nitrogen within cover crop. Means were compared using a least significant difference (LSD) method. The effects of agriculture practices on N cycling microbes were visualized using scatterplots made by graphing software Origin Pro 2019b.

Pearson correlation analyses were used to determine correlation among genes, gene transcripts, transcript/gene ratios, and soil properties by rcorr function of package Hmisc in R 3.6.1 (the R Foundation for Statistical Computing). Correlation coefficient *P* values were then adjusted with a correction for multiple comparisons using the BH method of the Hmisc package. A heatmap based on Pearson correlations was used to visualize the significant positive and negative correlations among measured soil properties, N cycle gene abundance, gene transcript abundance, and transcript/gene ratios, and was performed by the pheatmap package in R 3.6.1.

Nonmetric multidimensional scaling (NMDS) based on Bray-Curtis distances was performed in R 3.6.1 with packages vegan 2.5-5 and phyloseq to reveal the distribution patterns of N cycling functional groups and their activity among seasons and management practices. Analysis of similarities (ANOSIM), a rank-based nonparametric statistical test, was also performed by R with the function anosim in package vegan to compare groups and test the null hypothesis that the similarity between groups is greater than or equal to the similarity within the groups. Dispersion indices were calculated in R with the function betadisper in package vegan to assess the multivariate homogeneity of group dispersions. The function TukeyHSD.betadisper in package vegan was used to calculate Tukey’s honest significant differences between groups. In this study, statistically significant difference was accepted at a *P* value of <0.05 unless otherwise noted.
