# Supplementary figures and images for: Soil Health Management Enhances Microbial Nitrogen Cycling Capacity and Activity
Source: mSphere. 2021 Jan 13;6(1):e01237-20. doi: 10.1128/mSphere.01237-20 (PMC7845608; doi:10.1128/mSphere.01237-20)

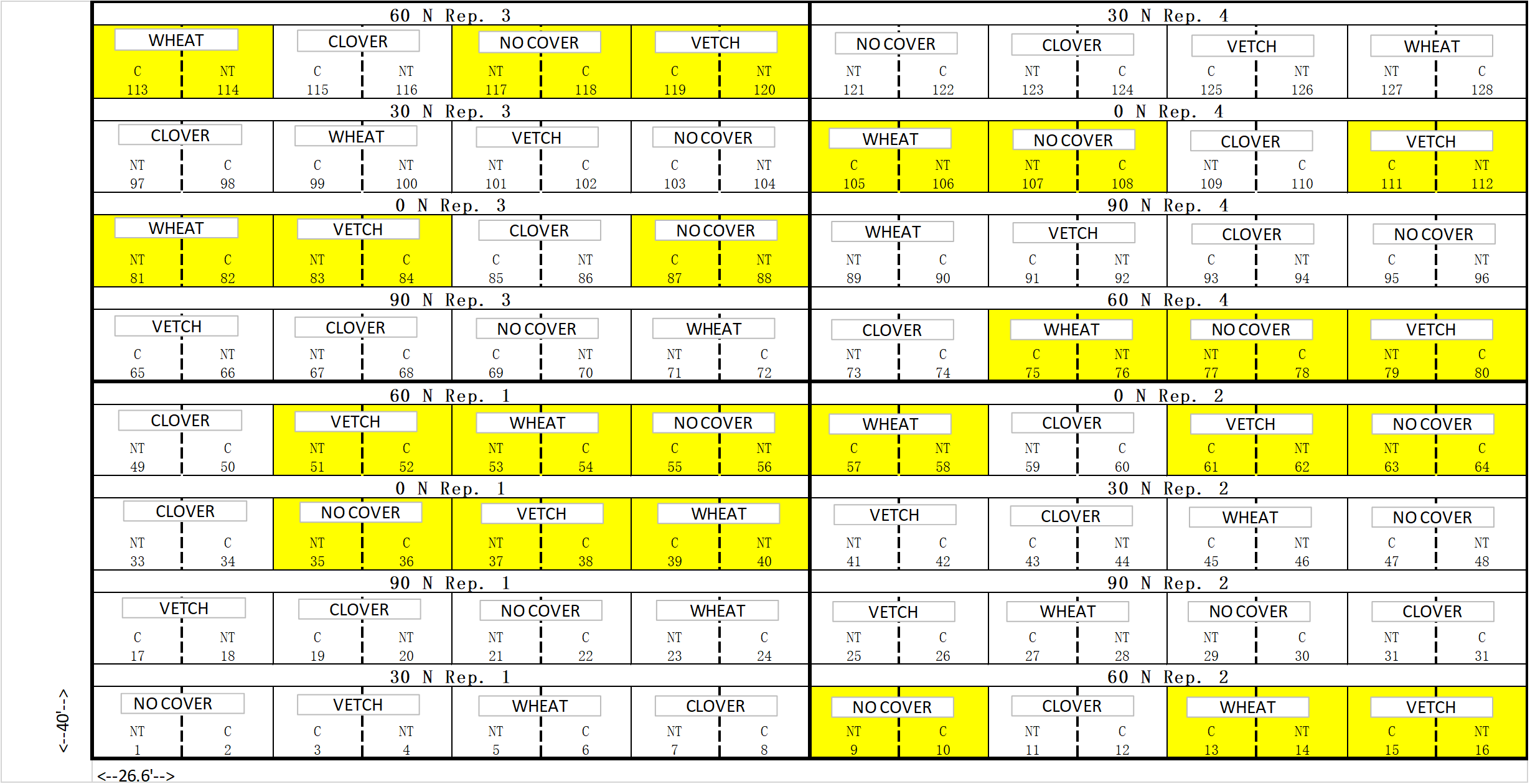

Supplement: FIG S1 [file mSphere.01237-20_sf001.tif]

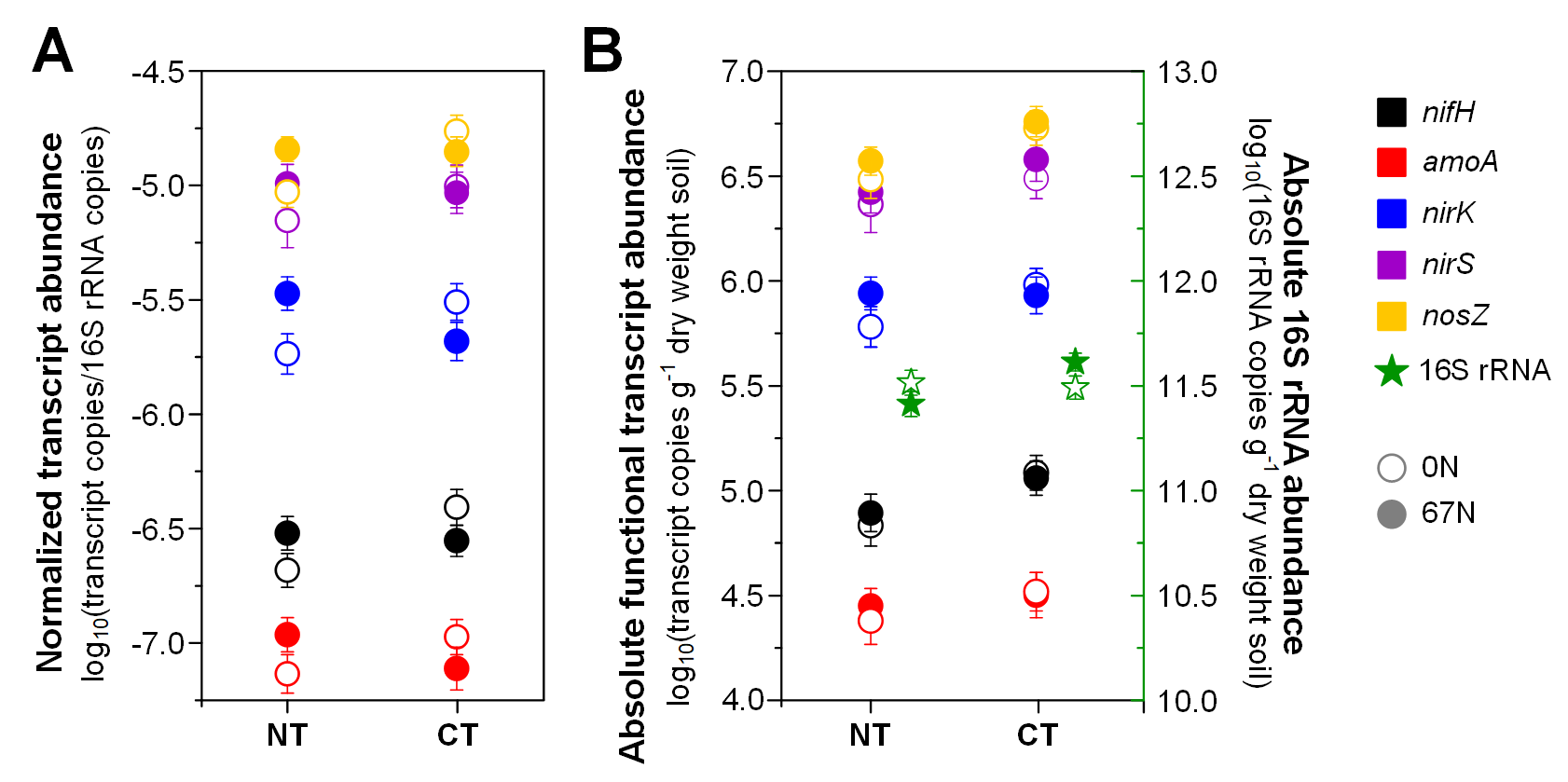

Supplement: FIG S2 [file mSphere.01237-20_sf002.tif]
