# Supplementary material for: Soil Health Management Enhances Microbial Nitrogen Cycling Capacity and Activity
Source: mSphere. 2021 Jan 13;6(1):e01237-20. doi: 10.1128/mSphere.01237-20 (PMC7845608; doi:10.1128/mSphere.01237-20)
Supplement: TEXT S1 [file mSphere.01237-20_s0001.docx]

**SUPPLEMENTAL METHODS** Description of methods used for soil properties measurements.

*From Li et al. "Impact of inorganic fertilizer, cover crops, tillage, and seasonality on soil N loss and retention" (in preparation).*

***METHOD S1 Bulk soil total N and C, soil pH, and soil water content***

A subsample of 2 mm-sieved fresh soil was oven dried at 65 °C, ground to powder, and analyzed for total N and total C using dry combustion (Stable Isotope Facility; University of California-Davis). A subsample of fresh soil was added to sterile water (1:2 extraction ratio), vortexed, and allowed to settle before measuring for pH (Ultrabasic, Denver Instrument, Bohemia, NY, USA). Another subsample of fresh soil was weighed, then heated at 105 ℃ for 48 hours and weighed again. The difference in weight was considered to be the weight of the water in the samples and the gravimetric soil water content (SWC) was calculated as SWC (%) = [(fresh weight soil – dry weight soil)/dry weight soil] × 100%.

***METHOD S2 Total extractable C, total extractable N, ammonium, and nitrate measurement***

For total extractable C (TEC) and total extractable N (TEN) measurement, 10 g field moist soil was extracted in 40 ml of 0.5M K_2_SO_4_ extract solution by shaking on a reciprocating shaker at 150 rpm for 4 h. The solution was then centrifuged at 3803 rcf for 5 minutes and filtered through a Whatman GF/B filter (0.9 to 1.2 µm pore size) into clean container to collect the filtrate. Filtered extracts were kept under –20℃ until further analysis. Soil microbial biomass N (MBN) and microbial biomass C (MBC) were measured by a chloroform slurry method (1). For each field sample, one soil subsample was chloroform-exposed and another subsample non-chloroform-exposed, then both were extracted with 0.5 M K_2_SO_4_ as described above. Filtered extracts were kept under –20℃ until further analysis. Both TEC and TEN concentrations in chloroform-exposed and non-chloroform-exposed soil subsamples were measured using an Organic Carbon analyzer modified with a nitrogen chemiluminescence detector (CLD) for combustion analysis (Aurora 1030W, OI Analytical, College Station, Taxes, USA). Non-chloroform-exposed soil extracts were analyzed for ammonium and nitrate using microplate-based spectrophotometric determinations (2, 3). Soil extracts were pipetted into 96-well plates. Reagents were added to each well. After reactions complete, the plate was read on a microplate reader (Synergy HT, BioTek, Winooski, VT, USA).

***METHOD S3 Incubation for net N mineralization and nitrification rates***

Fresh soils were incubated in mason jars at 25 ℃ for 7 days. The incubated soils were extracted and analyzed for soil ammonium and nitrate concentrations as described in Method S2 to determine post-incubation soil ammonium and nitrate concentrations (ammonium_post-incubation_ and nitrate_post-incubation_), which were then used for N transformation calculations described in Method S4.

***METHOD S4 Soil N and C pool and transformation rate calculations***

Soil total N (TN) and total C (TN) was evaluated as concentration (mg N or C g^−1^ soil). Soil microbial biomass N (MBN, μg N g^−1^ soil) concentration was calculated as MBN = TEN_chloroform-exposed_ – TEN_non-chloroform-exposed_. Soil microbial biomass C (MBC, μg C g^−1^ soil) concentration was calculated as MBC = TEC_chloroform-exposed_ – TEC_non-chloroform-exposed_. Potential N mineralization (PNM, μg N g^−1^ soil d^−1^) rate was calculated as PNM = (ammonium_post-incubation_ – ammonium_pre-incubation_)/7 day. Potential N nitrification (PNN, μg N g^−1^ soil d^−1^) rate was calculated as PNN = nitrate_post-incubation_ – nitrate_pre-incubation_)/7 day.

***METHOD S5 Gas sample collection and analysis***

Gas emissions were measured and calculated using standardized sampling designs and data processing protocols used by the USDA-ARS Greenhouse Reduction through Agricultural Carbon Enhancement Network (GRACEnet). Gas measurements were taken with static vented chambers using a stratified sampling design in which gases were sampled with syringes and injected into evacuated vials at four evenly spaced time-points over 30 minutes (0, 10, 20, 30 minutes). To account for diurnal variability, gas samples were collected at times of the day closely corresponding to the daily average temperature (midmorning, early evening). Environmental conditions, such as air temperature, soil temperature, and soil moisture, were also measured at the time of collection.

Concentrations of N_2_O in air samples from the sampling chamber headspace were measured at the USDA-ARS laboratory in Lincoln, NE using a gas chromatograph equipped with an electron capture detector. Soil gas emission rates were calculated as the change in headspace gas concentration over time within the enclosed chamber volume.

**REFERENCES**

1. Fierer N, Schimel J. 2002. Effects of drying-rewetting frequency on soil carbon and nitrogen transformations. Soil Biol Biochem 34:777-787.

2. Doane TA, Horwáth WR. 2003. Spectrophotometric determination of nitrate with a single reagent. Anal Lett 36:2713-2722.

3. Rhine ED, Mulvaney RL, Pratt EJ, Sims GK. 1998. Improving the Berthelot reaction for determining ammonium in soil extracts and water. Soil Sci Soc Am J 62:473-480.
