# Supplementary material for: Soil Health Management Enhances Microbial Nitrogen Cycling Capacity and Activity
Source: mSphere. 2021 Jan 13;6(1):e01237-20. doi: 10.1128/mSphere.01237-20 (PMC7845608; doi:10.1128/mSphere.01237-20)
Supplement: TABLE S1 [file mSphere.01237-20_st001.docx]

| Gene | Primers | Sequence (5'-3') | Reaction parameters |
| --- | --- | --- | --- |
| *amoA* (AOB^†^) | *amoA* 1F  *amoA* 2R | GGGGTTTCTACTGGTGGT  CCCCTCKGSAAAGCCTTCTTC | 95 ℃ for 3 min × 1 cycle; (94 ℃ for 1 min, 56 ℃ for 45 s, 72 ℃ for 1 min) × 40 cycles; 72 ℃ for 10 min × 1 cycle |
| *nifH* | IGK3  DVV | GCIWTHTAYGGIAARGGIGGIATHGGIAA  ATIGCRAAICCICCRCAIACIACRTC | 95 ℃ for 10 min × 1 cycle; (95 ℃ for 30 s, 58 ℃ for 1 min, 72 ℃ for 1 min) × 40 cycles; 72 ℃ for 10 min × 1 cycle |
| *nirS* | cd3aF  R3cd | GTSAACGTSAAGGARACSGG  GASTTCGGRTGSGTCTTGA | 95 ℃ for 10 min × 1 cycle; (94 ℃ for 30 s, 57 ℃ for 1 min, 72 ℃ for 1 min) × 40 cycles; 72 ℃ for 10 min × 1 cycle |
| *nirK* | F1aCu  R3Cu | ATCATGGTSCTGCCGCG  GCCTCGATCAGRTTGTGGTT | 94 ℃ for 2 min × 1 cycle; (94 ℃ for 30 s, 58 ℃ for 1 min, 72 ℃ for 1 min) × 40 cycles; 72 ℃ for 10 min × 1 cycle |
| *nosZ* | *nosZ*-I F  *nosZ*-I R | CGCRACGGCAASAAGGTSMSSGT  CAKRTGCAKSGCRTGGCAGAA | 94 ℃ for 5 min × 1 cycle; (94 ℃ for 40 s, 60 ℃ for 40 s, 72 ℃ for 1 min) × 40 cycles; 72 ℃ for 10 min × 1 cycle |

^†^AOB = Ammonia oxidizing bacteria
