# Supplementary material for: Soil Health Management Enhances Microbial Nitrogen Cycling Capacity and Activity
Source: mSphere. 2021 Jan 13;6(1):e01237-20. doi: 10.1128/mSphere.01237-20 (PMC7845608; doi:10.1128/mSphere.01237-20)
Supplement: TABLE S2 [file mSphere.01237-20_st002.docx]

| Factor | SWC^†^ | pH | NO_3_-N | NH_4_-N | TEC | TEN | TC | TN | MBC | MBN | C:N | N_2_O-N |
| --- | --- | --- | --- | --- | --- | --- | --- | --- | --- | --- | --- | --- |
| Season | **401.85^***^** | **17.90^***^** | **552.04^***^** | **34.86^***^** | **107.33^***^** | **265.32^***^** | **34.48^***^** | **31.34^***^** | **60.07^***^** | **13.01^***^** | **11.93^***^** | **15.27^***^** |
| Tillage (Till) | **8.52^**^** | 2.55 | 2.53 | 3.80 | **13.13^***^** | 0.16 | **18.94^***^** | **17.71^***^** | 1.77 | 0.01 | 3.53 | 0.74 |
| Cover | **5.43^**^** | 2.77 | **203.02^***^** | **7.45^***^** | **14.09^***^** | **96.02^***^** | **24.98^***^** | **46.25^***^** | **3.73^*^** | **5.05^**^** | 1.76 | **20.37^***^** |
| Nitrogen (N) | **12.67^***^** | 0.47 | **4.71^*^** | 0.24 | 3.51 | 0.56 | **47.47^***^** | **65.04^***^** | 0.90 | 0.16 | 1.54 | **5.49^*^** |
| Season*Till | 2.03 | 1.44 | 0.43 | **8.07^***^** | **2.85^*^** | 1.20 | **4.78^**^** | **4.20^**^** | **3.72^*^** | 1.84 | 0.87 | 0.55 |
| Season*Cover | 2.15 | 0.74 | **89.22^***^** | **8.61^***^** | 1.54 | **33.23^***^** | 0.84 | 0.30 | **2.26^*^** | 0.74 | 0.82 | **7.01^***^** |
| Season*N | 0.80 | 0.64 | 1.02 | 1.67 | 0.48 | 0.42 | 0.43 | 0.11 | 0.07 | **3.57^*^** | 0.55 | 1.78 |
| Till*Cover | 2.74 | 2.07 | 0.13 | 1.35 | 1.35 | 0.24 | 0.61 | 0.63 | 0.48 | 0.40 | 0.25 | 1.28 |
| Till*N | 0.31 | 2.90 | 0.02 | 2.13 | 0.05 | 1.18 | 1.04 | 1.03 | 0.40 | 0.16 | 0.09 | 1.01 |
| Cover*N | 1.22 | **7.67^***^** | 0.35 | 2.32 | 0.88 | 0.06 | **3.70^*^** | **3.94^*^** | 0.42 | 1.44 | 1.13 | 0.22 |
| Till*Cover*N | 1.56 | 0.61 | 0.62 | 3.06 | 0.01 | 0.51 | 0.62 | 0.54 | 1.76 | 0.45 | 0.09 | 3.06 |
| Season*Till*Cover | 0.51 | 0.49 | 1.00 | 0.88 | 0.65 | 0.19 | 0.68 | 0.45 | 0.41 | 1.53 | 0.61 | 1.18 |
| Season*Till*N | 0.24 | 0.81 | 2.05 | 0.15 | 0.09 | 0.36 | 1.27 | 0.66 | 0.16 | 1.78 | 0.54 | 0.88 |
| Season*Cover*N | 0.30 | 0.91 | 0.27 | 0.83 | 0.71 | 0.50 | 0.61 | 0.31 | 1.08 | 1.56 | 0.40 | 1.66 |
| Season*Till*Cover*N | 2.22 | 0.23 | 0.33 | 0.46 | 0.17 | 0.35 | 1.49 | 1.37 | 1.61 | 1.40 | 0.68 | 0.58 |

**TABLE S2** Continued.

| Factor | Field net nitrification | Field net N mineralization | Incubated net nitrification | Incubated net N mineralization |
| --- | --- | --- | --- | --- |
| Season | **501.86^***^** | **455.53^***^** | **19.91^***^** | **7.32^***^** |
| Tillage (Till) | 0.48 | 1.62 | 0.61 | 0.46 |
| Cover | **32.47^***^** | **28.08^***^** | **29.27^***^** | **20.06^***^** |
| Nitrogen (N) | 0.01 | 0.35 | **6.68^*^** | **10.11^**^** |
| Season*Till | 1.01 | 1.11 | 1.54 | 1.59 |
| Season*Cover | **80.63^***^** | **53.22^***^** | **6.92^***^** | **3.33^**^** |
| Season*N | 1.29 | 0.36 | **5.10^**^** | 1.98 |
| Till*Cover | 0.35 | 0.78 | 0.66 | 0.10 |
| Till*N | 2.53 | 2.08 | 2.51 | 0.11 |
| Cover*N | 0.98 | 0.53 | 0.85 | 0.58 |
| Till*Cover*N | 0.17 | 0.31 | 0.15 | 0.74 |
| Season*Till*Cover | 1.38 | 1.82 | 1.53 | 0.51 |
| Season*Till*N | 2.43 | 1.68 | 1.41 | 0.57 |
| Season*Cover*N | 0.50 | 0.63 | 1.42 | 0.68 |
| Season*Till*Cover*N | 0.27 | 0.81 | 0.25 | 0.38 |

Significance level: * *p*-value ≤ 0.05; ** *p*-value ≤ 0.01; *** *p*-value ≤ 0.001.

^†^SWC: soil water content; TEC: total extractable C; TEN: total extractable N; TC: total C; TN: total N; MBC: microbial biomass C; MBN: microbial biomass N.
