# Supplementary material for: Soil Health Management Enhances Microbial Nitrogen Cycling Capacity and Activity
Source: mSphere. 2021 Jan 13;6(1):e01237-20. doi: 10.1128/mSphere.01237-20 (PMC7845608; doi:10.1128/mSphere.01237-20)
Supplement: TABLE S3 [file mSphere.01237-20_st003.docx]

| Treatment^†^ | | SWC^‡^ | pH | NO_3_-N | NH_4_-N | TEC | TEN | TC | TN | MBC | MBN | C:N | N_2_O-N | Nitrification | | N Mineralization | |
| --- | --- | --- | --- | --- | --- | --- | --- | --- | --- | --- | --- | --- | --- | --- | --- | --- | --- |
|  |  |  |  |  |  |  |  |  |  |  |  |  |  | Field | Incubate | Field | Incubate |
| Season | Apr | 0.17^c^ | 5.76^c^ | 3.43^b^ | 1.40^c^ | 128.70^a^ | 8.08^b^ | 9.97^c^ | 1.15^c^ | 1139.07^a^ | 31.00^b^ | 8.62^c^ | 0.45^b^ | -0.05^c^ | 0.12^b^ | -0.04^b^ | 0.14^b^ |
|  | May | 0.19^b^ | 5.93^b^ | 14.53^a^ | 1.65^b^ | 53.96^c^ | 27.17^a^ | 11.35^b^ | 1.29^b^ | 431.33^b^ | 39.35^a^ | 8.80^bc^ | 1.18^a^ | 0.27^a^ | 0.39^a^ | 0.27^a^ | 0.32^a^ |
|  | Oct | 0.05^d^ | 6.27^a^ | 2.32^c^ | 2.02^a^ | 97.46^b^ | 9.54^b^ | 13.48^a^ | 1.40^a^ | 508.45^b^ | 20.12^c^ | 9.54^a^ | 1.35^a^ | -0.09^d^ | 0.10^b^ | -0.09^c^ | 0.12^b^ |
|  | Nov | 0.23^a^ | 6.23^a^ | 1.99^c^ | 0.81^d^ | 45.74^c^ | 5.81^c^ | 10.16^c^ | 1.12^c^ | 426.65^b^ | 27.05^b^ | 9.07^b^ | 0.68^b^ | -0.01^b^ | 0.17^b^ | -0.03^b^ | 0.19^b^ |
| Tillage | NT | 0.15^b^ | 6.00 | 5.38 | 1.55 | 88.26^a^ | 12.87 | 11.84^a^ | 1.29^a^ | 600.39 | 29.39 | 9.12 | 0.96 | 0.03 | 0.19 | 0.02 | 0.18 |
|  | CT | 0.17^a^ | 6.09 | 5.85 | 1.38 | 74.68^b^ | 12.49 | 10.64^b^ | 1.19^b^ | 653.32 | 29.58 | 8.90 | 0.87 | 0.03 | 0.21 | 0.03 | 0.20 |
| Cover | NC | 0.15^b^ | 6.07 | 4.03^b^ | 1.30^b^ | 74.26^b^ | 10.14^b^ | 10.49^b^ | 1.17^b^ | 589.13^b^ | 25.74^b^ | 8.89 | 0.68^b^ | 0.02^b^ | 0.11^b^ | 0.02^b^ | 0.12^b^ |
|  | V | 0.16^a^ | 5.96 | 9.16^a^ | 1.70^a^ | 95.53^a^ | 18.51^a^ | 12.61^a^ | 1.40^a^ | 709.26^a^ | 33.33^a^ | 8.98 | 1.40^a^ | 0.07^a^ | 0.36^a^ | 0.07^a^ | 0.34^a^ |
|  | W | 0.17^a^ | 6.12 | 3.55^c^ | 1.41^b^ | 74.61^b^ | 9.34^b^ | 10.62^b^ | 1.15^b^ | 581.88^b^ | 29.20^b^ | 9.15 | 0.66^b^ | 0.00^b^ | 0.12^b^ | 0.00^b^ | 0.12^b^ |
| Nitrogen | 0N | 0.15^b^ | 6.01 | 5.36^b^ | 1.43 | 77.16 | 12.38 | 10.19^b^ | 1.13^b^ | 644.81 | 29.00 | 8.84 | 0.78^b^ | 0.03 | 0.16^b^ | 0.02 | 0.14^b^ |
|  | 67N | 0.17^a^ | 6.03 | 5.80^a^ | 1.49 | 84.97 | 12.84 | 12.19^a^ | 1.34^a^ | 602.64 | 29.65 | 9.08 | 1.04^a^ | 0.03 | 0.24^a^ | 0.03 | 0.24^a^ |
| Season  *  Tillage | Apr-NT | 0.17 | 5.66 | 3.10 | 1.42^b^ | 138.25^a^ | 7.76 | 10.32^cd^ | 1.19^de^ | 983.93^b^ | 26.98 | 8.63 | 0.58 | -0.06 | 0.10 | -0.06 | 0.13 |
|  | Apr-CT | 0.17 | 5.87 | 3.78 | 1.38^b^ | 119.16^b^ | 8.39 | 9.62^c^ | 1.10^e^ | 1294.21^a^ | 34.84 | 8.62 | 0.33 | -0.04 | 0.14 | -0.03 | 0.16 |
|  | May-NT | 0.18 | 5.93 | 14.11 | 1.57^b^ | 56.78^d^ | 26.78 | 11.94^b^ | 1.34^b^ | 418.48^c^ | 42.37 | 8.93 | 1.13 | 0.26 | 0.34 | 0.26 | 0.25 |
|  | May-CT | 0.20 | 5.93 | 14.94 | 1.72^b^ | 51.14^d^ | 27.56 | 10.77^c^ | 1.24^cd^ | 444.19^c^ | 36.33 | 8.68 | 1.23 | 0.27 | 0.45 | 0.27 | 0.39 |
|  | Oct-NT | 0.04 | 6.30 | 2.22 | 2.47^a^ | 111.78^b^ | 10.41 | 14.90^a^ | 1.52^a^ | 555.48^c^ | 21.44 | 9.79 | 1.37 | -0.09 | 0.10 | -0.08 | 0.12 |
|  | Oct-CT | 0.05 | 6.25 | 2.43 | 1.57^b^ | 83.14^c^ | 8.68 | 12.05^b^ | 1.29^bc^ | 463.39^c^ | 18.73 | 9.29 | 1.34 | -0.09 | 0.10 | -0.09 | 0.12 |
|  | Nov-NT | 0.23 | 6.13 | 1.95 | 0.75^c^ | 46.21^d^ | 6.30 | 10.18^cd^ | 1.12^e^ | 441.82^c^ | 26.13 | 9.12 | 0.77 | 0.00 | 0.21 | -0.04 | 0.22 |
|  | Nov-CT | 0.24 | 6.33 | 2.03 | 0.86^c^ | 45.27^d^ | 5.32 | 10.14^cd^ | 1.13^e^ | 411.48^c^ | 27.97 | 9.01 | 0.58 | -0.01 | 0.14 | -0.02 | 0.15 |
| Season  *  Cover | Apr-NC | 0.16 | 5.74 | 2.63^ef^ | 0.81^e^ | 118.18 | 7.43^ef^ | 9.15 | 1.08 | 975.97^b^ | 26.87 | 8.43 | 0.40^c^ | -0.05^ef^ | -0.09^g^ | -0.05^ef^ | 0.01^fg^ |
|  | Apr-V | 0.18 | 5.71 | 4.78^d^ | 2.33^a^ | 155.08 | 10.75^cd^ | 10.80 | 1.28 | 1390.12^a^ | 37.90 | 8.44 | 0.55^c^ | -0.03^de^ | 0.51^a^ | -0.01^d^ | 0.44^ab^ |
|  | Apr-W | 0.18 | 5.84 | 2.85^ef^ | 1.07^de^ | 112.85 | 6.03^efg^ | 9.96 | 1.09 | 1051.12^b^ | 26.96 | 9.00 | 0.42^c^ | -0.07^f^ | -0.05^fg^ | -0.07^f^ | -0.03^g^ |
|  | May-NC | 0.18 | 6.02 | 10.00^b^ | 1.77^bc^ | 49.68 | 19.49^b^ | 10.80 | 1.22 | 439.70^cd^ | 35.85 | 8.83 | 0.53^c^ | 0.18^b^ | 0.31^b^ | 0.20^b^ | 0.22^cde^ |
|  | May-V | 0.18 | 5.69 | 25.39^a^ | 1.39^cd^ | 63.81 | 43.45^a^ | 12.60 | 1.46 | 398.47^cd^ | 41.20 | 8.67 | 2.40^a^ | 0.49^a^ | 0.56^a^ | 0.46^a^ | 0.49^a^ |
|  | May-W | 0.21 | 6.07 | 8.19^c^ | 1.78^bc^ | 48.39 | 18.57^b^ | 10.66 | 1.19 | 455.82^cd^ | 40.99 | 8.91 | 0.61^c^ | 0.13^c^ | 0.31^bc^ | 0.14^c^ | 0.25^cd^ |
|  | Oct-NC | 0.04 | 6.28 | 1.67^fg^ | 1.82^b^ | 86.30 | 8.03^def^ | 12.62 | 1.34 | 482.56^cd^ | 15.08 | 9.33 | 1.38^b^ | -0.06^f^ | 0.05^efg^ | -0.06^f^ | 0.14^defg^ |
|  | Oct-V | 0.06 | 6.25 | 3.62^de^ | 2.17^ab^ | 110.27 | 11.67^c^ | 15.28 | 1.58 | 612.53^c^ | 23.25 | 9.64 | 1.86^b^ | -0.16^g^ | 0.17^bcde^ | -0.15^g^ | 0.13^defg^ |
|  | Oct-W | 0.05 | 6.29 | 1.58^g^ | 2.06^ab^ | 95.81 | 8.93^cde^ | 12.53 | 1.29 | 425.06^cd^ | 21.94 | 9.64 | 0.82^c^ | -0.05^ef^ | 0.07^def^ | -0.05^def^ | 0.08^efg^ |
|  | Nov-NC | 0.23 | 6.23 | 1.82^fg^ | 0.80^e^ | 42.87 | 5.45^fg^ | 9.40 | 1.05 | 458.30^cd^ | 24.62 | 8.97 | 0.42^c^ | 0.00^d^ | 0.16^cde^ | -0.02^de^ | 0.10^defg^ |
|  | Nov-V | 0.24 | 6.18 | 2.86^ef^ | 0.89^e^ | 52.95 | 8.17^def^ | 11.76 | 1.29 | 435.91^cd^ | 30.96 | 9.19 | 0.80^c^ | -0.03^de^ | 0.20^bcd^ | -0.03^def^ | 0.31^bc^ |
|  | Nov-W | 0.23 | 6.28 | 1.29^g^ | 0.73^e^ | 41.40 | 3.81^g^ | 9.32 | 1.04 | 385.73^cd^ | 25.57 | 9.05 | 0.80^c^ | 0.00^d^ | 0.16^cde^ | -0.03^def^ | 0.15^cdef^ |
| Season  *  Nitrogen | Apr-0N | 0.16 | 5.83 | 3.57 | 1.23 | 121.78 | 7.64 | 8.90 | 1.05 | 1146.55 | 25.13^de^ | 8.46 | 0.35 | -0.05 | 0.00^f^ | -0.04 | 0.02 |
|  | Apr-67N | 0.18 | 5.70 | 3.30 | 1.58 | 135.63 | 8.51 | 11.04 | 1.25 | 1131.59 | 36.61^bc^ | 8.78 | 0.56 | -0.05 | 0.25^bc^ | -0.04 | 0.26 |
|  | May-0N | 0.18 | 5.89 | 14.21 | 1.73 | 49.96 | 27.45 | 10.65 | 1.21 | 468.34 | 44.07^a^ | 8.84 | 1.25 | 0.26 | 0.34^ab^ | 0.26 | 0.26 |
|  | May-67N | 0.20 | 5.97 | 14.84 | 1.56 | 57.96 | 26.89 | 12.06 | 1.37 | 394.32 | 34.63^b^ | 8.76 | 1.12 | 0.27 | 0.45^a^ | 0.27 | 0.38 |
|  | Oct-0N | 0.05 | 6.32 | 1.80 | 2.00 | 96.45 | 9.41 | 12.39 | 1.31 | 539.85 | 21.53^de^ | 9.42 | 1.08 | -0.09 | 0.08^ef^ | -0.09 | 0.11 |
|  | Oct-67N | 0.05 | 6.23 | 2.80 | 2.04 | 98.47 | 9.68 | 14.57 | 1.50 | 478.36 | 18.77^e^ | 9.66 | 1.62 | -0.09 | 0.12^de^ | -0.08 | 0.13 |
|  | Nov-0N | 0.22 | 6.23 | 1.71 | 0.82 | 43.64 | 5.35 | 9.24 | 1.03 | 447.00 | 25.37^cde^ | 9.02 | 0.48 | 0.00 | 0.21^cd^ | -0.03 | 0.16 |
|  | Nov-67N | 0.24 | 6.23 | 2.27 | 0.79 | 47.84 | 6.28 | 11.08 | 1.22 | 406.30 | 28.73^bcd^ | 9.11 | 0.87 | -0.02 | 0.14^cde^ | -0.03 | 0.22 |
| Cover  *  Nitrogen | NC-0N | 0.14 | 6.04^bc^ | 3.78 | 1.37 | 68.41 | 10.04 | 9.47^c^ | 1.08^d^ | 638.84 | 27.72 | 8.71 | 0.59 | 0.02 | 0.09 | 0.02 | 0.08 |
|  | NC-67N | 0.16 | 6.10^ab^ | 4.28 | 1.23 | 80.10 | 10.24 | 11.52^b^ | 1.26^c^ | 539.42 | 23.69 | 9.07 | 0.78 | 0.02 | 0.12 | 0.01 | 0.15 |
|  | V-0N | 0.16 | 5.87^c^ | 8.74 | 1.55 | 90.92 | 18.18 | 12.16^ab^ | 1.34^b^ | 719.95 | 31.22 | 9.01 | 1.30 | 0.07 | 0.32 | 0.06 | 0.29 |
|  | V-67N | 0.17 | 6.04^bc^ | 9.59 | 1.84 | 100.14 | 18.84 | 13.06^a^ | 1.46^a^ | 698.57 | 35.44 | 8.95 | 1.51 | 0.07 | 0.40 | 0.07 | 0.39 |
|  | W-0N | 0.16 | 6.30^a^ | 3.55 | 1.43 | 74.55 | 9.24 | 9.26^c^ | 1.02^d^ | 594.21 | 28.91 | 9.09 | 0.48 | 0.00 | 0.06 | -0.01 | 0.04 |
|  | W-67N | 0.18 | 5.94^bc^ | 3.55 | 1.39 | 74.68 | 9.43 | 11.97^b^ | 1.29^bc^ | 569.95 | 29.45 | 9.21 | 0.84 | 0.01 | 0.18 | 0.01 | 0.19 |

^†^NC = no cover; V = vetch; W = wheat; NT = no tillage; CT = conventional tillage; 0N = no fertilization; 67N = 67 kg N ha^-1^ fertilization.

^‡^SWC = soil water content; TEC = total extractable carbon; TEN = total extractable nitrogen; TC = total soil carbon; TN = total soil nitrogen.

Note: the unit for SWC is g H_2_O g^-1^ dry weight soil (g H_2_O gdw^-1^); unit for NO_3_-N, NH_4_-N, TEN and MBN is μg N gdw^-1^; unit for TN is mg N gdw^-1^; unit for TEC and MBC is μg C gdw^-1^; unit for TC is mg C gdw^-1^; unit for nitrification and N mineralization is μg N gdw^-1^d^-1^; unit for N_2_O-N is g N ha^-1^d^-1^.
