# Supplementary material for: Soil Health Management Enhances Microbial Nitrogen Cycling Capacity and Activity
Source: mSphere. 2021 Jan 13;6(1):e01237-20. doi: 10.1128/mSphere.01237-20 (PMC7845608; doi:10.1128/mSphere.01237-20)
Supplement: TABLE S4 [file mSphere.01237-20_st004.docx]

| Treatment^†^ | | *nifH* | | AOB^‡^ *amoA* | | *nirK* | | *nirS* | | *nosZ* | |
| --- | --- | --- | --- | --- | --- | --- | --- | --- | --- | --- | --- |
|  |  | Gene | Transcript | Gene | Transcript | Gene | Transcript | Gene | Transcript | Gene | Transcript |
| Season | April | -3.799^a^ | -6.676^b^ | -4.153^b^ | -7.164^b^ | -2.396^a^ | -5.420^b^ | -1.855^b^ | -5.160^b^ | -2.334^b^ | -4.901^b^ |
|  | May | -4.088^c^ | -6.795^b^ | -4.077^b^ | -7.039^b^ | -2.575^b^ | -5.895^c^ | -2.005^c^ | -5.478^c^ | -2.472^c^ | -4.800^b^ |
|  | October | -4.178^c^ | -6.721^b^ | -3.805^a^ | -7.125^b^ | -2.421^a^ | -5.878^c^ | -1.837^b^ | -5.182^b^ | -2.024^a^ | -4.655^a^ |
|  | November | -3.944^b^ | -5.969^a^ | -4.507^c^ | -6.856^a^ | -2.358^a^ | -5.207^a^ | -1.705^a^ | -4.361^a^ | -2.327^b^ | -5.131^c^ |
| Cover | NC | -4.065 | -6.538^ab^ | -4.389^c^ | -7.030^b^ | -2.464 | -5.485^a^ | -1.900 | -4.988^a^ | -2.334^b^ | -4.810^a^ |
|  | V | -4.003 | -6.461^a^ | -3.785^a^ | -6.696^a^ | -2.431 | -5.466^a^ | -1.819 | -4.879^a^ | -2.216^a^ | -4.722^a^ |
|  | W | -3.939 | -6.622^b^ | -4.233^b^ | -7.411^c^ | -2.418 | -5.849^b^ | -1.832 | -5.269^b^ | -2.317^b^ | -5.082^b^ |
| Tillage | NT | -4.079^b^ | -6.601^b^ | -4.132 | -7.049 | -2.428 | -5.604 | -1.890^b^ | -5.072 | -2.300 | -4.935^b^ |
|  | CT | -3.925^a^ | -6.480^a^ | -4.140 | -7.042 | -2.448 | -5.595 | -1.811^a^ | -5.019 | -2.279 | -4.808^a^ |
| Nitrogen | 0N | -4.004 | -6.545 | -4.128 | -7.054 | -2.459 | -5.623 | -1.917^b^ | -5.079 | -2.351^b^ | -4.896 |
|  | 67N | -4.001 | -6.537 | -4.143 | -7.038 | -2.416 | -5.577 | -1.784^a^ | -5.012 | -2.227^a^ | -4.847 |
| Tillage  *  Nitrogen | NT-0N | -4.099 | -6.683^c^ | -4.141 | -7.135^b^ | -2.459 | -5.736^b^ | -1.959 | -5.153 | -2.365 | -5.029^b^ |
|  | NT-67N | -4.059 | -6.520^ab^ | -4.123 | -6.964^a^ | -2.396 | -5.472^a^ | -1.821 | -4.991 | -2.234 | -4.842^a^ |
|  | CT-0N | -3.908 | -6.406^a^ | -4.115 | -6.974^a^ | -2.459 | -5.509^a^ | -1.874 | -5.004 | -2.337 | -4.763^a^ |
|  | CT-67N | -3.943 | -6.554^b^ | -4.164 | -7.111^b^ | -2.436 | -5.682^b^ | -1.747 | -5.033 | -2.220 | -4.853^a^ |
| Season  *  Cover | Apr-NC | -3.857 | -6.622^b^ | -4.457 | -7.175^cd^ | -2.383 | -5.242^b^ | -1.875 | -5.131^ef^ | -2.378 | -4.849^bc^ |
|  | Apr-V | -3.821 | -6.701^bc^ | -3.755 | -6.836^b^ | -2.391 | -5.339^bc^ | -1.826 | -4.81^cd^ | -2.237 | -4.763^b^ |
|  | Apr-W | -3.718 | -6.707^bc^ | -4.248 | -7.482^ef^ | -2.415 | -5.679^de^ | -1.863 | -5.539^gh^ | -2.386 | -5.090^d^ |
|  | May-NC | -4.094 | -6.965^d^ | -4.306 | -7.361^def^ | -2.578 | -6.055^fg^ | -2.078 | -5.643^h^ | -2.547 | -5.024^cd^ |
|  | May-V | -4.056 | -6.638^b^ | -3.761 | -6.508^a^ | -2.578 | -5.636^de^ | -1.932 | -5.290^fg^ | -2.376 | -4.512^a^ |
|  | May-W | -4.115 | -6.783^bcd^ | -4.165 | -7.247^cde^ | -2.568 | -5.992^f^ | -2.004 | -5.502^gh^ | -2.494 | -4.865^bc^ |
|  | Oct-NC | -4.358 | -6.646^b^ | -4.131 | -7.083^c^ | -2.485 | -5.753^e^ | -1.918 | -5.113^ef^ | -2.011 | -4.548^a^ |
|  | Oct-V | -4.187 | -6.615^b^ | -3.425 | -6.729^ab^ | -2.386 | -5.640^de^ | -1.818 | -4.924^de^ | -1.994 | -4.550^a^ |
|  | Oct-W | -3.990 | -6.903^cd^ | -3.860 | -7.562^f^ | -2.394 | -6.242^g^ | -1.775 | -5.510^gh^ | -2.068 | -4.866^bc^ |
|  | Nov-NC | -3.953 | -5.920^a^ | -4.661 | -6.499^a^ | -2.412 | -4.890^a^ | -1.729 | -4.067^a^ | -2.401 | -4.819^b^ |
|  | Nov-V | -3.947 | -5.891^a^ | -4.202 | -6.713^ab^ | -2.367 | -5.249^bc^ | -1.701 | -4.492^b^ | -2.256 | -5.064^d^ |
|  | Nov-W | -3.931 | -6.097^a^ | -4.659 | -7.355^def^ | -2.296 | -5.482^cd^ | -1.686 | -4.524^bc^ | -2.322 | -5.509^e^ |
| Season  *  Tillage | Apr-NT | -3.909 | -6.669^bc^ | -4.193^c^ | -7.218^cd^ | -2.419 | -5.470 | -1.939 | -5.091 | -2.374 | -5.001^cd^ |
|  | Apr-CT | -3.689 | -6.684^bc^ | -4.114^bc^ | -7.110^bcd^ | -2.373 | -5.370 | -1.771 | -5.228 | -2.294 | -4.801^b^ |
|  | May-NT | -4.167 | -6.931^d^ | -4.133^bc^ | -7.018^b^ | -2.556 | -5.873 | -2.035 | -5.535 | -2.511 | -4.863^bc^ |
|  | May-CT | -4.010 | -6.660^b^ | -4.021^b^ | -7.059^bcd^ | -2.594 | -5.916 | -1.974 | -5.421 | -2.433 | -4.737^b^ |
|  | Oct-NT | -4.303 | -6.841^cd^ | -3.802^a^ | -7.223^d^ | -2.417 | -5.870 | -1.905 | -5.236 | -2.030 | -4.787^b^ |
|  | Oct-CT | -4.054 | -6.601^b^ | -3.809^a^ | -7.027^bc^ | -2.426 | -5.886 | -1.769 | -5.129 | -2.018 | -4.523^a^ |
|  | Nov-NT | -3.938 | -5.964^a^ | -4.399^d^ | -6.738^a^ | -2.319 | -5.204 | -1.681 | -4.425 | -2.283 | -5.091^de^ |
|  | Nov-CT | -3.950 | -5.975^a^ | -4.615^e^ | -6.973^b^ | -2.398 | -5.210 | -1.729 | -4.297 | -2.370 | -5.171^e^ |
| Cover  *  Nitrogen | NC-0N | -4.071 | -6.628^bc^ | -4.435^d^ | -7.233^c^ | -2.519 | -5.563^a^ | -1.995^b^ | -5.088^bc^ | -2.416 | -4.943^b^ |
|  | NC-67N | -4.060 | -6.448^a^ | -4.343^d^ | -6.826^b^ | -2.4109 | -5.407^a^ | -1.805^a^ | -4.888^ab^ | -2.252 | -4.677^a^ |
|  | V-0N | -4.022 | -6.443^a^ | -3.865^b^ | -6.633^a^ | -2.4309 | -5.438^a^ | -1.82^a^ | -4.755^a^ | -2.256 | -4.660^a^ |
|  | V-67N | -3.98 | -6.479^ab^ | -3.706^a^ | -6.760^ab^ | -2.431 | -5.494^a^ | -1.817^a^ | -5.003^bc^ | -2.176 | -4.785^a^ |
|  | W-0N | -3.919 | -6.562^abc^ | -4.085^c^ | -7.296^c^ | -2.429 | -5.866^b^ | -1.934^b^ | -5.393^d^ | -2.381 | -5.085^c^ |
|  | W-67N | -3.959 | -6.682^c^ | -4.381^d^ | -7.527^d^ | -2.407 | -5.831^b^ | -1.731^a^ | -5.145^c^ | -2.254 | -5.080^bc^ |
| Season  *  Cover  *  Tillage | Apr-NC-NT | -3.915 | -6.567^defg^ | -4.596 | -7.063^efg^ | -2.394 | -5.200^bc^ | -1.937 | -5.052^def^ | -2.408 | -4.752^cdefg^ |
|  | Apr-NC-CT | -3.799 | -6.676^fgh^ | -4.318 | -7.287^fgh^ | -2.371 | -5.284^bcde^ | -1.814 | -5.209^efgh^ | -2.349 | -4.946^gh^ |
|  | Apr-V-NT | -3.973 | -6.820^ghi^ | -3.743 | -6.970^def^ | -2.400 | -5.445^bcdef^ | -1.944 | -4.564^bc^ | -2.281 | -4.935^fgh^ |
|  | Apr-V-CT | -3.669 | -6.582^defg^ | -3.766 | -6.702^bcd^ | -2.382 | -5.233^bcd^ | -1.708 | -5.056^def^ | -2.194 | -4.592^bcd^ |
|  | Apr-W-NT | -3.839 | -6.620^efgh^ | -4.239 | -7.622^ij^ | -2.464 | -5.765^fghi^ | -1.936 | -5.657^ij^ | -2.433 | -5.316^j^ |
|  | Apr-W-CT | -3.598 | -6.793^fghi^ | -4.256 | -7.341^ghi^ | -2.365 | -5.593^efgh^ | -1.790 | -5.420^fghi^ | -2.338 | -4.864^defgh^ |
|  | May-NC-NT | -4.162 | -7.266^j^ | -4.368 | -7.439^hij^ | -2.504 | -6.239^kl^ | -2.095 | -5.950^j^ | -2.546 | -5.232^ij^ |
|  | May-NC-CT | -4.026 | -6.664^fgh^ | -4.244 | -7.283^fgh^ | -2.652 | -5.870^hij^ | -2.060 | -5.335^fghi^ | -2.548 | -4.816^cdefg^ |
|  | May-V-NT | -4.200 | -6.631^efgh^ | -3.839 | -6.396^ab^ | -2.601 | -5.542^defgh^ | -1.932 | -5.199^efgh^ | -2.425 | -4.451^ab^ |
|  | May-V-CT | -3.913 | -6.646^fgh^ | -3.682 | -6.619^bc^ | -2.555 | -5.730^fghi^ | -1.933 | -5.381^fghi^ | -2.327 | -4.572^bc^ |
|  | May-W-NT | -4.140 | -6.895^hi^ | -4.194 | -7.218^fgh^ | -2.562 | -5.836^hij^ | -2.078 | -5.458^fghi^ | -2.563 | -4.907^efgh^ |
|  | May-W-CT | -4.090 | -6.671^fgh^ | -4.136 | -7.276^fgh^ | -2.574 | -6.148^jk^ | -1.930 | -5.547^ghij^ | -2.425 | -4.823^cdefg^ |
|  | Oct-NC-NT | -4.591 | -6.810^ghi^ | -4.204 | -7.448^hij^ | -2.494 | -5.832^hij^ | -2.003 | -5.111^ef^ | -2.009 | -4.637^bcde^ |
|  | Oct-NC-CT | -4.125 | -6.483^def^ | -4.059 | -6.718^bcd^ | -2.476 | -5.674^fghi^ | -1.834 | -5.115^ef^ | -2.014 | -4.459^ab^ |
|  | Oct-V-NT | -4.218 | -6.896^hi^ | -3.492 | -6.858^cde^ | -2.350 | -5.794^ghi^ | -1.865 | -5.154^efg^ | -1.975 | -4.815^cdefg^ |
|  | Oct-V-CT | -4.156 | -6.334^cde^ | -3.357 | -6.601^bc^ | -2.422 | -5.485^cdefg^ | -1.770 | -4.693^cd^ | -2.014 | -4.285^a^ |
|  | Oct-W-NT | -4.100 | -6.818^ghi^ | -3.709 | -7.363^ghi^ | -2.407 | -5.985^ijk^ | -1.848 | -5.443^fghi^ | -2.107 | -4.908^efgh^ |
|  | Oct-W-CT | -3.880 | -6.987^ij^ | -4.011 | -7.762^j^ | -2.380 | -6.498^l^ | -1.703 | -5.578^hij^ | -2.028 | -4.824^cdefg^ |
|  | Nov-NC-NT | -3.998 | -5.752^a^ | -4.569 | -6.223^a^ | -2.426 | -4.532^a^ | -1.793 | -3.880^a^ | -2.415 | -4.659^bcdef^ |
|  | Nov-NC-CT | -3.908 | -6.088^bc^ | -4.753 | -6.775^cde^ | -2.398 | -5.249^bcd^ | -1.664 | -4.253^ab^ | -2.387 | -4.979^ghi^ |
|  | Nov-V-NT | -3.958 | -5.865^ab^ | -4.063 | -6.611^bc^ | -2.313 | -5.242^bcd^ | -1.674 | -4.563^bc^ | -2.206 | -5.000^ghi^ |
|  | Nov-V-CT | -3.936 | -5.918^ab^ | -4.341 | -6.814^cde^ | -2.421 | -5.256^bcd^ | -1.729 | -4.421^bc^ | -2.307 | -5.129^hij^ |
|  | Nov-W-NT | -3.857 | -6.276^cd^ | -4.565 | -7.381^ghi^ | -2.217 | -5.839^hij^ | -1.576 | -4.832^cde^ | -2.230 | -5.613^k^ |
|  | Nov-W-CT | -4.006 | -5.918^ab^ | -4.752 | -7.330^ghi^ | -2.375 | -5.124^b^ | -1.795 | -4.216^ab^ | -2.415 | -5.405^jk^ |
| Season  *  Cover  *  Nitrogen | Apr-NC-0N | -3.915 | -6.739^cd^ | -4.596 | -7.283^hi^ | -2.394 | -5.268^bcde^ | -1.937 | -5.204 | -2.408 | -4.879^defghi^ |
|  | Apr-NC-67N | -3.973 | -6.504^c^ | -3.743 | -7.067^efgh^ | -2.400 | -5.216^bcd^ | -1.944 | -5.058 | -2.281 | -4.819^defgh^ |
|  | Apr-V-0N | -3.839 | -6.752^cd^ | -4.239 | -6.804^bcde^ | -2.464 | -5.331^cdef^ | -1.936 | -4.490 | -2.433 | -4.754^defg^ |
|  | Apr-V-67N | -3.799 | -6.649^cd^ | -4.318 | -6.868^cdef^ | -2.371 | -5.347^cdef^ | -1.814 | -5.130 | -2.349 | -4.773^defg^ |
|  | Apr-W-0N | -3.669 | -6.708^cd^ | -3.766 | -7.723^k^ | -2.382 | -5.862^hijk^ | -1.708 | -5.744 | -2.194 | -5.183^jk^ |
|  | Apr-W-67N | -3.598 | -6.705^cd^ | -4.256 | -7.240^ghi^ | -2.365 | -5.495^cdefg^ | -1.790 | -5.333 | -2.338 | -4.998^ghij^ |
|  | May-NC-0N | -4.162 | -7.080^ef^ | -4.368 | -7.570^ijk^ | -2.504 | -6.169^kl^ | -2.095 | -5.778 | -2.546 | -5.111^ijk^ |
|  | May-NC-67N | -4.200 | -6.850^de^ | -3.839 | -7.152^fgh^ | -2.601 | -5.941^ijk^ | -1.932 | -5.508 | -2.425 | -4.936^fghij^ |
|  | May-V-0N | -4.140 | -6.581^cd^ | -4.194 | -6.519^ab^ | -2.562 | -5.586^efgh^ | -2.078 | -5.230 | -2.563 | -4.398^ab^ |
|  | May-V-67N | -4.026 | -6.696^cd^ | -4.244 | -6.496^ab^ | -2.652 | -5.687^ghij^ | -2.060 | -5.350 | -2.548 | -4.625^bcd^ |
|  | May-W-0N | -3.913 | -6.737^cd^ | -3.682 | -7.314^hij^ | -2.555 | -5.873^hijk^ | -1.933 | -5.586 | -2.327 | -4.834^defghi^ |
|  | May-W-67N | -4.090 | -6.829^de^ | -4.136 | -7.179^fgh^ | -2.574 | -6.111^kl^ | -1.930 | -5.419 | -2.425 | -4.896^defghi^ |
|  | Oct-NC-0N | -4.591 | -6.838^de^ | -4.204 | -7.350^hij^ | -2.494 | -5.998^jk^ | -2.003 | -5.271 | -2.009 | -4.863^defghi^ |
|  | Oct-NC-67N | -4.218 | -6.455^bc^ | -3.492 | -6.817^bcde^ | -2.350 | -5.509^cdefg^ | -1.865 | -4.956 | -1.975 | -4.233^a^ |
|  | Oct-V-0N | -4.100 | -6.540^cd^ | -3.709 | -6.701^bc^ | -2.407 | -5.523^defg^ | -1.848 | -4.844 | -2.107 | -4.415^abc^ |
|  | Oct-V-67N | -4.125 | -6.689^cd^ | -4.059 | -6.758^bcde^ | -2.476 | -5.756^ghij^ | -1.834 | -5.003 | -2.014 | -4.685^cdef^ |
|  | Oct-W-0N | -4.156 | -6.642^cd^ | -3.357 | -7.055^defgh^ | -2.422 | -6.109^kl^ | -1.770 | -5.501 | -2.014 | -4.647^bcde^ |
|  | Oct-W-67N | -3.880 | -7.163^f^ | -4.011 | -8.069^l^ | -2.380 | -6.374^l^ | -1.703 | -5.520 | -2.028 | -5.084^hijk^ |
|  | Nov-NC-0N | -3.998 | -5.856^a^ | -4.569 | -6.730^bcd^ | -2.426 | -4.818^a^ | -1.793 | -4.101 | -2.415 | -4.917^efghij^ |
|  | Nov-NC-67N | -3.958 | -5.984^a^ | -4.063 | -6.268^a^ | -2.313 | -4.962^ab^ | -1.674 | -4.033 | -2.206 | -4.721^defg^ |
|  | Nov-V-0N | -3.857 | -5.899^a^ | -4.565 | -6.508^ab^ | -2.217 | -5.314^cdef^ | -1.576 | -4.457 | -2.230 | -5.073^hijk^ |
|  | Nov-V-67N | -3.908 | -5.883^a^ | -4.753 | -6.917^cdefg^ | -2.398 | -5.184^bc^ | -1.664 | -4.527 | -2.387 | -5.056^hij^ |
|  | Nov-W-0N | -3.936 | -6.162^ab^ | -4.341 | -7.092^efgh^ | -2.421 | -5.621^fghi^ | -1.729 | -4.740 | -2.307 | -5.676^l^ |
|  | Nov-W-67N | -4.006 | -6.032^a^ | -4.752 | -7.619^jk^ | -2.375 | -5.342^cdef^ | -1.795 | -4.308 | -2.415 | -5.342^k^ |
| Season  *  Tillage  *  Cover  *  Nitrogen | Apr-NT-NC-0N | -3.738 | -6.818 | -4.716 | -7.380 | -2.367 | -5.341 | -1.995 | -5.237 | -2.470 | -4.956 |
|  | Apr-NT-NC-67N | -4.091 | -6.316 | -4.476 | -6.745 | -2.422 | -5.058 | -1.878 | -4.868 | -2.345 | -4.548 |
|  | Apr-NT-V-0N | -4.141 | -6.886 | -3.892 | -6.940 | -2.364 | -5.526 | -1.904 | -3.889 | -2.254 | -4.917 |
|  | Apr-NT-V-67N | -3.805 | -6.753 | -3.594 | -6.999 | -2.435 | -5.364 | -1.984 | -5.239 | -2.308 | -4.954 |
|  | Apr-NT-W-0N | -3.938 | -6.638 | -3.931 | -7.927 | -2.536 | -6.069 | -2.098 | -5.922 | -2.493 | -5.375 |
|  | Apr-NT-W-67N | -3.739 | -6.602 | -4.547 | -7.318 | -2.391 | -5.460 | -1.773 | -5.393 | -2.373 | -5.258 |
|  | Apr-CT-NC-0N | -3.717 | -6.659 | -4.266 | -7.185 | -2.352 | -5.196 | -1.880 | -5.171 | -2.388 | -4.803 |
|  | Apr-CT-NC-67N | -3.881 | -6.693 | -4.371 | -7.389 | -2.390 | -5.373 | -1.748 | -5.247 | -2.310 | -5.089 |
|  | Apr-CT-V-0N | -3.620 | -6.618 | -3.752 | -6.668 | -2.321 | -5.136 | -1.684 | -5.090 | -2.136 | -4.591 |
|  | Apr-CT-V-67N | -3.719 | -6.546 | -3.780 | -6.737 | -2.443 | -5.330 | -1.732 | -5.022 | -2.252 | -4.592 |
|  | Apr-CT-W-0N | -3.632 | -6.778 | -4.150 | -7.520 | -2.356 | -5.655 | -1.906 | -5.566 | -2.466 | -4.991 |
|  | Apr-CT-W-67N | -3.563 | -6.809 | -4.362 | -7.162 | -2.375 | -5.530 | -1.675 | -5.273 | -2.211 | -4.738 |
|  | May-NT-NC-0N | -4.147 | -7.499 | -4.522 | -7.826 | -2.596 | -6.389 | -2.196 | -6.156 | -2.616 | -5.423 |
|  | May-NT-NC-67N | -4.177 | -7.033 | -4.213 | -7.052 | -2.412 | -6.090 | -1.995 | -5.744 | -2.476 | -5.041 |
|  | May-NT-V-0N | -4.192 | -6.700 | -3.920 | -6.504 | -2.559 | -5.581 | -1.904 | -5.254 | -2.472 | -4.409 |
|  | May-NT-V-67N | -4.208 | -6.562 | -3.757 | -6.288 | -2.644 | -5.504 | -1.960 | -5.143 | -2.379 | -4.493 |
|  | May-NT-W-0N | -4.051 | -6.807 | -4.044 | -7.276 | -2.547 | -5.723 | -2.133 | -5.528 | -2.587 | -4.855 |
|  | May-NT-W-67N | -4.228 | -6.983 | -4.343 | -7.159 | -2.577 | -5.950 | -2.024 | -5.387 | -2.538 | -4.958 |
|  | May-CT-NC-0N | -4.019 | -6.662 | -4.287 | -7.314 | -2.683 | -5.949 | -2.037 | -5.399 | -2.556 | -4.800 |
|  | May-CT-NC-67N | -4.032 | -6.666 | -4.200 | -7.253 | -2.621 | -5.792 | -2.082 | -5.272 | -2.540 | -4.832 |
|  | May-CT-V-0N | -3.886 | -6.461 | -3.688 | -6.535 | -2.560 | -5.591 | -1.839 | -5.205 | -2.328 | -4.387 |
|  | May-CT-V-67N | -3.940 | -6.831 | -3.677 | -6.704 | -2.551 | -5.870 | -2.027 | -5.556 | -2.326 | -4.758 |
|  | May-CT-W-0N | -3.992 | -6.667 | -4.069 | -7.352 | -2.542 | -6.024 | -2.011 | -5.645 | -2.440 | -4.812 |
|  | May-CT-W-67N | -4.189 | -6.675 | -4.202 | -7.199 | -2.605 | -6.273 | -1.848 | -5.450 | -2.410 | -4.833 |
|  | Oct-NT-NC-0N | -4.654 | -7.111 | -4.045 | -7.717 | -2.584 | -6.216 | -2.077 | -5.239 | -2.064 | -5.043 |
|  | Oct-NT-NC-67N | -4.528 | -6.509 | -4.362 | -7.179 | -2.404 | -5.448 | -1.929 | -4.984 | -1.953 | -4.231 |
|  | Oct-NT-V-0N | -4.408 | -6.875 | -3.773 | -6.907 | -2.396 | -5.881 | -1.938 | -5.222 | -2.095 | -4.723 |
|  | Oct-NT-V-67N | -4.029 | -6.917 | -3.212 | -6.808 | -2.303 | -5.707 | -1.792 | -5.087 | -1.855 | -4.908 |
|  | Oct-NT-W-0N | -4.014 | -6.620 | -3.676 | -7.063 | -2.508 | -6.064 | -1.934 | -5.654 | -2.186 | -4.862 |
|  | Oct-NT-W-67N | -4.186 | -7.017 | -3.741 | -7.662 | -2.306 | -5.906 | -1.761 | -5.232 | -2.028 | -4.953 |
|  | Oct-CT-NC-0N | -4.343 | -6.564 | -4.215 | -6.982 | -2.641 | -5.780 | -2.082 | -5.303 | -2.227 | -4.683 |
|  | Oct-CT-NC-67N | -3.907 | -6.402 | -3.903 | -6.455 | -2.310 | -5.569 | -1.585 | -4.927 | -1.800 | -4.236 |
|  | Oct-CT-V-0N | -4.119 | -6.206 | -3.422 | -6.494 | -2.486 | -5.165 | -1.837 | -4.466 | -2.165 | -4.108 |
|  | Oct-CT-V-67N | -4.193 | -6.462 | -3.292 | -6.707 | -2.357 | -5.805 | -1.703 | -4.919 | -1.862 | -4.463 |
|  | Oct-CT-W-0N | -3.901 | -6.665 | -3.945 | -7.048 | -2.404 | -6.153 | -1.849 | -5.348 | -2.129 | -4.432 |
|  | Oct-CT-W-67N | -3.859 | -7.309 | -4.077 | -8.475 | -2.356 | -6.842 | -1.557 | -5.809 | -1.928 | -5.215 |
|  | Nov-NT-NC-0N | -4.039 | -5.782 | -4.641 | -6.384 | -2.470 | -4.482 | -1.899 | -3.946 | -2.517 | -4.726 |
|  | Nov-NT-NC-67N | -3.958 | -5.722 | -4.498 | -6.062 | -2.383 | -4.583 | -1.687 | -3.814 | -2.312 | -4.591 |
|  | Nov-NT-V-0N | -4.050 | -5.969 | -4.172 | -6.451 | -2.382 | -5.288 | -1.809 | -4.505 | -2.355 | -5.072 |
|  | Nov-NT-V-67N | -3.866 | -5.761 | -3.954 | -6.770 | -2.243 | -5.197 | -1.538 | -4.622 | -2.056 | -4.928 |
|  | Nov-NT-W-0N | -3.820 | -6.487 | -4.357 | -7.240 | -2.202 | -6.276 | -1.624 | -5.284 | -2.271 | -5.988 |
|  | Nov-NT-W-67N | -3.893 | -6.064 | -4.773 | -7.522 | -2.233 | -5.402 | -1.528 | -4.380 | -2.190 | -5.237 |
|  | Nov-CT-NC-0N | -3.909 | -5.930 | -4.784 | -7.076 | -2.457 | -5.155 | -1.795 | -4.256 | -2.493 | -5.107 |
|  | Nov-CT-NC-67N | -3.908 | -6.246 | -4.722 | -6.474 | -2.340 | -5.342 | -1.533 | -4.251 | -2.282 | -4.850 |
|  | Nov-CT-V-0N | -3.756 | -5.830 | -4.300 | -6.565 | -2.373 | -5.340 | -1.658 | -4.409 | -2.247 | -5.073 |
|  | Nov-CT-V-67N | -4.115 | -6.006 | -4.382 | -7.064 | -2.470 | -5.171 | -1.799 | -4.433 | -2.368 | -5.184 |
|  | Nov-CT-W-0N | -4.001 | -5.836 | -4.504 | -6.944 | -2.334 | -4.967 | -1.913 | -4.196 | -2.474 | -5.365 |
|  | Nov-CT-W-67N | -4.011 | -5.999 | -5.001 | -7.716 | -2.416 | -5.282 | -1.677 | -4.236 | -2.355 | -5.446 |

Note: letters that represent the significant differences for the combination effects of season, tillage, cover crop, and nitrogen were not shown due to table space limitation.

^†^NC = no cover; V = vetch; W = wheat; NT = no tillage; CT = conventional tillage; 0N = no fertilization; 67N = 67 kg N ha^-1^ fertilization.

^‡^AOB = Ammonia oxidizing bacteria
