# Supplementary material for: Soil Health Management Enhances Microbial Nitrogen Cycling Capacity and Activity
Source: mSphere. 2021 Jan 13;6(1):e01237-20. doi: 10.1128/mSphere.01237-20 (PMC7845608; doi:10.1128/mSphere.01237-20)
Supplement: TABLE S5 [file mSphere.01237-20_st005.docx]

| Treatment^†^ | | *nifH* | | AOB^‡^ *amoA* | | *nirK* | | *nirS* | | *nosZ* | | 16S | |
| --- | --- | --- | --- | --- | --- | --- | --- | --- | --- | --- | --- | --- | --- |
|  |  | Gene | Transcript | Gene | Transcript | Gene | Transcript | Gene | Transcript | Gene | Transcript | Gene | Transcript |
| Season | April | 5.503^a^ | 4.579^c^ | 5.149^c^ | 4.091^c^ | 6.906^c^ | 5.835^b^ | 7.447^c^ | 6.095^c^ | 6.968^c^ | 6.354^c^ | 9.302^c^ | 11.255^c^ |
|  | May | 5.465^a^ | 4.731^c^ | 5.477^b^ | 4.488^b^ | 6.979^b^ | 5.632^c^ | 7.549^b^ | 6.048^c^ | 7.081^b^ | 6.727^b^ | 9.554^a^ | 11.527^b^ |
|  | October | 5.220^b^ | 4.953^b^ | 5.593^a^ | 4.550^ab^ | 6.977^b^ | 5.796^b^ | 7.561^b^ | 6.492^b^ | 7.374^a^ | 7.020^a^ | 9.398^b^ | 11.675^a^ |
|  | November | 5.505^a^ | 5.608^a^ | 4.941^d^ | 4.718^a^ | 7.090^a^ | 6.371^a^ | 7.743^a^ | 7.217^a^ | 7.122^b^ | 6.444^c^ | 9.448^b^ | 11.575^ab^ |
| Tillage | NT | 5.292^b^ | 4.863^b^ | 5.239^b^ | 4.414 | 6.943^b^ | 5.861 | 7.481^b^ | 6.393^b^ | 7.071^b^ | 6.528^b^ | 9.371^b^ | 11.463 |
|  | CT | 5.555^a^ | 5.072^a^ | 5.340^a^ | 4.510 | 7.032^a^ | 5.957 | 7.669^a^ | 6.533^a^ | 7.201^a^ | 6.744^a^ | 9.480^a^ | 11.552 |
| Cover | NC | 5.371 | 4.852^b^ | 5.047^c^ | 4.358^b^ | 6.972 | 5.906^b^ | 7.536^b^ | 6.403^b^ | 7.102^b^ | 6.578^b^ | 9.436 | 11.388^b^ |
|  | V | 5.434 | 5.156^a^ | 5.651^a^ | 4.921^a^ | 7.006 | 6.152^a^ | 7.617^a^ | 6.739^a^ | 7.221^a^ | 6.895^a^ | 9.437 | 11.618^a^ |
|  | W | 5.465 | 4.895^b^ | 5.171^b^ | 4.106^c^ | 6.986 | 5.669^c^ | 7.572^ab^ | 6.248^b^ | 7.086^b^ | 6.435^c^ | 9.404 | 11.517^a^ |
| Nitrogen | 0N | 5.436^a^ | 4.946 | 5.341^a^ | 4.431 | 7.001 | 5.867 | 7.542^b^ | 6.429 | 7.126 | 6.610 | 9.466^a^ | 11.512 |
|  | 67N | 5.385^b^ | 4.976 | 5.243^b^ | 4.475 | 6.970 | 5.936 | 7.602^a^ | 6.501 | 7.159 | 6.665 | 9.386^b^ | 11.513 |
| Season  *  Tillage | Apr-NT | 5.349 | 4.498^d^ | 5.065^c^ | 3.949 | 6.838 | 5.697 | 7.319 | 6.075 | 6.884 | 6.166^d^ | 9.258 | 11.166 |
|  | Apr-CT | 5.657 | 4.659^cd^ | 5.232^b^ | 4.233 | 6.973 | 5.973 | 7.575 | 6.114 | 7.052 | 6.542^c^ | 9.346 | 11.343 |
|  | May-NT | 5.317 | 4.490^d^ | 5.351^b^ | 4.403 | 6.928 | 5.547 | 7.449 | 5.885 | 6.973 | 6.557^c^ | 9.484 | 11.420 |
|  | May-CT | 5.614 | 4.973^b^ | 5.603^a^ | 4.574 | 7.029 | 5.717 | 7.649 | 6.212 | 7.190 | 6.896^b^ | 9.623 | 11.633 |
|  | Oct-NT | 5.071 | 4.858^bc^ | 5.572^a^ | 4.476 | 6.957 | 5.829 | 7.468 | 6.463 | 7.343 | 6.913^b^ | 9.374 | 11.699 |
|  | Oct-CT | 5.368 | 5.048^b^ | 5.613^a^ | 4.623 | 6.996 | 5.764 | 7.653 | 6.521 | 7.404 | 7.127^a^ | 9.422 | 11.650 |
|  | Nov-NT | 5.430 | 5.608^a^ | 4.969^c^ | 4.827 | 7.049 | 6.371 | 7.687 | 7.150 | 7.084 | 6.476^c^ | 9.368 | 11.568 |
|  | Nov-CT | 5.579 | 5.608^a^ | 4.914^c^ | 4.609 | 7.131 | 6.372 | 7.800 | 7.285 | 7.159 | 6.411^c^ | 9.529 | 11.582 |
| Season  *  Cover | Apr-NC | 5.438 | 4.334 | 4.837 | 3.781^f^ | 6.912 | 5.714^efg^ | 7.419 | 5.825^i^ | 6.916 | 6.107^e^ | 9.294 | 10.955^d^ |
|  | Apr-V | 5.510 | 4.827 | 5.576 | 4.691^bc^ | 6.940 | 6.189^bc^ | 7.505 | 6.717^de^ | 7.094 | 6.764^bc^ | 9.331 | 11.527^b^ |
|  | Apr-W | 5.562 | 4.575 | 5.032 | 3.800^f^ | 6.866 | 5.603^fgh^ | 7.417 | 5.743^i^ | 6.894 | 6.191^e^ | 9.280 | 11.281^c^ |
|  | May-NC | 5.427 | 4.552 | 5.215 | 4.156^e^ | 6.942 | 5.462^gh^ | 7.443 | 5.874^hi^ | 6.974 | 6.493^d^ | 9.520 | 11.517^b^ |
|  | May-V | 5.463 | 4.887 | 5.759 | 5.018^a^ | 6.941 | 5.890^de^ | 7.587 | 6.236^fg^ | 7.143 | 7.014^ab^ | 9.519 | 11.526^b^ |
|  | May-W | 5.506 | 4.754 | 5.456 | 4.290^de^ | 7.053 | 5.545^gh^ | 7.617 | 6.035^ghi^ | 7.127 | 6.672^cd^ | 9.621 | 11.537^b^ |
|  | Oct-NC | 5.097 | 4.955 | 5.324 | 4.518^cd^ | 6.970 | 5.848^def^ | 7.537 | 6.488^ef^ | 7.444 | 7.053^a^ | 9.455 | 11.601^ab^ |
|  | Oct-V | 5.228 | 5.144 | 5.990 | 5.029^a^ | 7.030 | 6.119^cd^ | 7.598 | 6.835^cd^ | 7.421 | 7.208^a^ | 9.415 | 11.759^a^ |
|  | Oct-W | 5.333 | 4.761 | 5.463 | 4.102^ef^ | 6.930 | 5.422^h^ | 7.548 | 6.153^gh^ | 7.256 | 6.798^bc^ | 9.323 | 11.664^ab^ |
|  | Nov-NC | 5.521 | 5.566 | 4.813 | 4.977^ab^ | 7.062 | 6.600^a^ | 7.746 | 7.424^a^ | 7.073 | 6.660^cd^ | 9.474 | 11.480^bc^ |
|  | Nov-V | 5.534 | 5.767 | 5.279 | 4.946^ab^ | 7.114 | 6.410^ab^ | 7.780 | 7.167^ab^ | 7.224 | 6.594^cd^ | 9.481 | 11.659^ab^ |
|  | Nov-W | 5.459 | 5.490 | 4.732 | 4.231^de^ | 7.094 | 6.105^cd^ | 7.705 | 7.062^bc^ | 7.068 | 6.077^e^ | 9.390 | 11.586^ab^ |
| Season  *  Nitrogen | Apr-0N | 5.550 | 4.500 | 5.229 | 3.963 | 6.965 | 5.746 | 7.436 | 6.087 | 6.979 | 6.294^f^ | 9.347 | 11.232 |
|  | Apr-67N | 5.457 | 4.657 | 5.068 | 4.218 | 6.847 | 5.924 | 7.458 | 6.103 | 6.956 | 6.414^ef^ | 9.256 | 11.277 |
|  | May-0N | 5.515 | 4.799 | 5.474 | 4.464 | 6.981 | 5.722 | 7.542 | 6.067 | 7.063 | 6.817^bc^ | 9.562 | 11.598 |
|  | May-67N | 5.416 | 4.664 | 5.479 | 4.513 | 6.977 | 5.542 | 7.555 | 6.030 | 7.100 | 6.636^cd^ | 9.545 | 11.455 |
|  | Oct-0N | 5.244 | 4.992 | 5.638 | 4.630 | 6.981 | 5.789 | 7.531 | 6.460 | 7.340 | 7.024^a^ | 9.484 | 11.666 |
|  | Oct-67N | 5.195 | 4.914 | 5.547 | 4.469 | 6.972 | 5.804 | 7.590 | 6.524 | 7.407 | 7.016^ab^ | 9.312 | 11.683 |
|  | Nov-0N | 5.535 | 5.546 | 5.005 | 4.735 | 7.095 | 6.270 | 7.681 | 7.089 | 7.072 | 6.292^f^ | 9.464 | 11.514 |
|  | Nov-67N | 5.474 | 5.669 | 4.878 | 4.701 | 7.085 | 6.473 | 7.805 | 7.346 | 7.172 | 6.596^de^ | 9.433 | 11.636 |
| Tillage  *  Nitrogen | NT-0N | 5.332 | 4.833 | 5.291 | 4.378 | 6.972 | 5.781 | 7.472 | 6.364 | 7.066 | 6.484 | 9.431 | 11.514^ab^ |
|  | NT-67N | 5.251 | 4.893 | 5.188 | 4.449 | 6.914 | 5.941 | 7.489 | 6.422 | 7.076 | 6.571 | 9.310 | 11.413^b^ |
|  | CT-0N | 5.590 | 5.085 | 5.383 | 4.518 | 7.039 | 5.982 | 7.624 | 6.487 | 7.161 | 6.729 | 9.498 | 11.491^ab^ |
|  | CT-67N | 5.519 | 5.059 | 5.298 | 4.501 | 7.026 | 5.931 | 7.715 | 6.580 | 7.242 | 6.759 | 9.462 | 11.613^a^ |
| Cover  *  Nitrogen | NC-0N | 5.436 | 4.764 | 5.072^c^ | 4.154^cd^ | 6.988^b^ | 5.831 | 7.511^bc^ | 6.306^cd^ | 7.090^cd^ | 6.446^c^ | 9.506 | 11.389 |
|  | NC-67N | 5.305 | 4.939 | 5.023^c^ | 4.562^b^ | 6.955^b^ | 5.981 | 7.561^b^ | 6.499^bc^ | 7.113^bcd^ | 6.710^b^ | 9.366 | 11.388 |
|  | V-0N | 5.475 | 5.147 | 5.631^a^ | 4.957^a^ | 7.066^a^ | 6.152 | 7.674^a^ | 6.835^a^ | 7.240^a^ | 6.930^a^ | 9.496 | 11.590 |
|  | V-67N | 5.393 | 5.166 | 5.671^a^ | 4.885^a^ | 6.946^b^ | 6.152 | 7.560^b^ | 6.643^ab^ | 7.201^ab^ | 6.860^ab^ | 9.377 | 11.645 |
|  | W-0N | 5.471 | 4.953 | 5.348^b^ | 4.320^c^ | 6.954^b^ | 5.688 | 7.448^c^ | 6.154^d^ | 7.017^d^ | 6.457^c^ | 9.383 | 11.535 |
|  | W-67N | 5.458 | 4.824 | 5.035^c^ | 3.979^d^ | 7.009^ab^ | 5.675 | 7.686^a^ | 6.361^cd^ | 7.162^abc^ | 6.426^c^ | 9.416 | 11.506 |
| Tillage  *  Cover  *  Nitrogen | NT-NC-0N | 5.327 | 4.525 | 4.991 | 3.991 | 6.967 | 5.726 | 7.430 | 6.188^ef^ | 7.055 | 6.284 | 9.472 | 11.321 |
|  | NT-NC-67N | 5.118 | 4.854 | 4.919 | 4.489 | 6.901 | 5.954 | 7.434 | 6.396^cde^ | 7.034 | 6.646 | 9.306 | 11.249 |
|  | NT-V-0N | 5.278 | 5.069 | 5.536 | 4.976 | 7.050 | 6.108 | 7.587 | 6.959^a^ | 7.182 | 6.896 | 9.476 | 11.677 |
|  | NT-V-67N | 5.295 | 5.040 | 5.642 | 4.821 | 6.865 | 6.095 | 7.453 | 6.515^bcd^ | 7.122 | 6.717 | 9.272 | 11.538 |
|  | NT-W-0N | 5.391 | 4.905 | 5.345 | 4.167 | 6.898 | 5.510 | 7.399 | 5.946^f^ | 6.962 | 6.273 | 9.347 | 11.543 |
|  | NT-W-67N | 5.342 | 4.786 | 5.002 | 4.037 | 6.977 | 5.773 | 7.582 | 6.355^de^ | 7.071 | 6.351 | 9.353 | 11.453 |
|  | CT-NC-0N | 5.545 | 5.003 | 5.153 | 4.317 | 7.008 | 5.936 | 7.593 | 6.424^cde^ | 7.126 | 6.608 | 9.541 | 11.457 |
|  | CT-NC-67N | 5.493 | 5.025 | 5.126 | 4.634 | 7.010 | 6.008 | 7.688 | 6.602^bcd^ | 7.192 | 6.775 | 9.425 | 11.527 |
|  | CT-V-0N | 5.671 | 5.225 | 5.726 | 4.938 | 7.082 | 6.196 | 7.762 | 6.711^abc^ | 7.298 | 6.964 | 9.517 | 11.504 |
|  | CT-V-67N | 5.491 | 5.292 | 5.700 | 4.950 | 7.027 | 6.209 | 7.668 | 6.770^ab^ | 7.281 | 7.003 | 9.483 | 11.753 |
|  | CT-W-0N | 5.554 | 5.027 | 5.268 | 4.298 | 7.027 | 5.814 | 7.516 | 6.325^de^ | 7.058 | 6.614 | 9.436 | 11.514 |
|  | CT-W-67N | 5.574 | 4.861 | 5.069 | 3.920 | 7.041 | 5.577 | 7.790 | 6.366^de^ | 7.253 | 6.500 | 9.479 | 11.559 |
| Season  *  Cover  *  Tillage | Apr-NC-NT | 5.299 | 4.052^j^ | 4.618 | 3.556^i^ | 6.819 | 5.419^ijk^ | 7.277 | 5.567^jk^ | 6.806 | 5.867 | 9.213 | 10.618 |
|  | Apr-NC-CT | 5.576 | 4.616^gh^ | 5.05 | 4.005^ghi^ | 7.004 | 6.008^defg^ | 7.561 | 6.083^fghi^ | 7.026 | 6.346 | 9.375 | 11.292 |
|  | Apr-V-NT | 5.321 | 4.733^efgh^ | 5.551 | 4.582^cdef^ | 6.894 | 6.107^cdef^ | 7.350 | 6.988^bc^ | 7.013 | 6.617 | 9.294 | 11.552 |
|  | Apr-V-CT | 5.699 | 4.921^defg^ | 5.602 | 4.800^abc^ | 6.986 | 6.270^bcde^ | 7.660 | 6.447^defg^ | 7.174 | 6.911 | 9.368 | 11.50 |
|  | Apr-W-NT | 5.426 | 4.709^efgh^ | 5.026 | 3.707^hi^ | 6.801 | 5.565^hij^ | 7.330 | 5.672^ijk^ | 6.832 | 6.013 | 9.265 | 11.329 |
|  | Apr-W-CT | 5.697 | 4.440^hij^ | 5.039 | 3.892^ghi^ | 6.930 | 5.641^ghi^ | 7.505 | 5.814^hijk^ | 6.957 | 6.369 | 9.295 | 11.233 |
|  | May-NC-NT | 5.249 | 4.192^ij^ | 5.043 | 4.019^gh^ | 6.907 | 5.218^jk^ | 7.315 | 5.508^k^ | 6.865 | 6.226 | 9.411 | 11.458 |
|  | May-NC-CT | 5.604 | 4.912^defg^ | 5.386 | 4.293^efg^ | 6.978 | 5.706^ghi^ | 7.570 | 6.241^efgh^ | 7.082 | 6.760 | 9.630 | 11.576 |
|  | May-V-NT | 5.244 | 4.709^efgh^ | 5.605 | 4.944^abc^ | 6.843 | 5.797^ghji^ | 7.512 | 6.141^fgh^ | 7.019 | 6.888 | 9.444 | 11.340 |
|  | May-V-CT | 5.682 | 5.066^cdef^ | 5.912 | 5.093^ab^ | 7.039 | 5.982^defg^ | 7.662 | 6.332^defg^ | 7.268 | 7.140 | 9.595 | 11.712 |
|  | May-W-NT | 5.457 | 4.568^ghi^ | 5.403 | 4.245^fg^ | 7.035 | 5.627^ghi^ | 7.519 | 6.005^ghij^ | 7.034 | 6.557 | 9.597 | 11.463 |
|  | May-W-CT | 5.555 | 4.940^defg^ | 5.510 | 4.335^defg^ | 7.071 | 5.463^ijk^ | 7.715 | 6.064^fghi^ | 7.220 | 6.788 | 9.645 | 11.611 |
|  | Oct-NC-NT | 4.854 | 4.817^efgh^ | 5.241 | 4.179^fg^ | 6.950 | 5.795^fghi^ | 7.441 | 6.516^def^ | 7.435 | 6.990 | 9.444 | 11.627 |
|  | Oct-NC-CT | 5.341 | 5.092^cde^ | 5.407 | 4.857^abc^ | 6.991 | 5.901^efgh^ | 7.633 | 6.460^defg^ | 7.453 | 7.116 | 9.466 | 11.575 |
|  | Oct-V-NT | 5.144 | 4.909^defg^ | 5.870 | 4.947^abc^ | 7.013 | 6.011^defg^ | 7.497 | 6.650^cde^ | 7.387 | 6.989 | 9.362 | 11.805 |
|  | Oct-V-CT | 5.312 | 5.379^bc^ | 6.111 | 5.112^ab^ | 7.047 | 6.227^bcde^ | 7.699 | 7.020^bc^ | 7.455 | 7.427 | 9.468 | 11.712 |
|  | Oct-W-NT | 5.215 | 4.847^efg^ | 5.606 | 4.303^efg^ | 6.908 | 5.680^ghi^ | 7.467 | 6.223^efgh^ | 7.208 | 6.758 | 9.315 | 11.666 |
|  | Oct-W-CT | 5.451 | 4.675^fgh^ | 5.320 | 3.900^ghi^ | 6.951 | 5.164^k^ | 7.629 | 6.084^fghi^ | 7.304 | 6.838 | 9.332 | 11.662 |
|  | Nov-NC-NT | 5.488 | 5.697^ab^ | 4.917 | 5.208^a^ | 7.060 | 6.927^a^ | 7.693 | 7.578^a^ | 7.072 | 6.776 | 9.486 | 11.438 |
|  | Nov-NC-CT | 5.554 | 5.435^bc^ | 4.710 | 4.747^bcde^ | 7.065 | 6.274^bcde^ | 7.798 | 7.269^ab^ | 7.075 | 6.544 | 9.463 | 11.522 |
|  | Nov-V-NT | 5.436 | 5.867^a^ | 5.331 | 5.121^ab^ | 7.081 | 6.490^bc^ | 7.720 | 7.169^ab^ | 7.188 | 6.732 | 9.394 | 11.732 |
|  | Nov-V-CT | 5.632 | 5.667^ab^ | 5.227 | 4.771^abcd^ | 7.146 | 6.329^bcd^ | 7.839 | 7.164^ab^ | 7.260 | 6.457 | 9.567 | 11.585 |
|  | Nov-W-NT | 5.366 | 5.258^cd^ | 4.658 | 4.153^fgh^ | 7.005 | 5.695^ghi^ | 7.647 | 6.702^cd^ | 6.993 | 5.921 | 9.223 | 11.534 |
|  | Nov-W-CT | 5.552 | 5.721^ab^ | 4.805 | 4.309^efg^ | 7.183 | 6.514^b^ | 7.763 | 7.422^ab^ | 7.143 | 6.234 | 9.558 | 11.639 |
| Season  *  Cover  *  Nitrogen | Apr-NC-0N | 5.629 | 4.151^k^ | 4.865 | 3.607^j^ | 6.997 | 5.621^hij^ | 7.419 | 5.686 | 6.927 | 6.011^jk^ | 9.356 | 10.889 |
|  | Apr-NC-67N | 5.246 | 4.517^jk^ | 4.809 | 3.955^hij^ | 6.826 | 5.806^defgh^ | 7.419 | 5.964 | 6.905 | 6.203^hij^ | 9.232 | 11.021 |
|  | Apr-V-0N | 5.510 | 4.695^ghij^ | 5.569 | 4.643^cdefg^ | 7.048 | 6.117^cde^ | 7.597 | 6.958 | 7.196 | 6.694^cdef^ | 9.391 | 11.447 |
|  | Apr-V-67N | 5.510 | 4.958^defghi^ | 5.584 | 4.739^bcdef^ | 6.832 | 6.260^abc^ | 7.414 | 6.477 | 6.991 | 6.834^bcd^ | 9.271 | 11.607 |
|  | Apr-W-0N | 5.509 | 4.653^hij^ | 5.254 | 3.638^ij^ | 6.849 | 5.499^hij^ | 7.293 | 5.617 | 6.815 | 6.178^ij^ | 9.295 | 11.361 |
|  | Apr-W-67N | 5.614 | 4.497^jk^ | 4.811 | 3.962^hij^ | 6.883 | 5.707^fghi^ | 7.542 | 5.869 | 6.973 | 6.204^ghij^ | 9.266 | 11.202 |
|  | May-NC-0N | 5.492 | 4.484^jk^ | 5.170 | 3.994^hij^ | 6.935 | 5.395^ij^ | 7.458 | 5.786 | 6.989 | 6.453^efghi^ | 9.575 | 11.564 |
|  | May-NC-67N | 5.361 | 4.620^ij^ | 5.259 | 4.318^fgh^ | 6.949 | 5.530^hij^ | 7.427 | 5.962 | 6.958 | 6.534^defghi^ | 9.466 | 11.470 |
|  | May-V-0N | 5.529 | 5.057^defg^ | 5.764 | 5.118^ab^ | 7.009 | 6.052^cdef^ | 7.696 | 6.408 | 7.168 | 7.240^a^ | 9.568 | 11.638 |
|  | May-V-67N | 5.397 | 4.718^fghij^ | 5.754 | 4.918^abcd^ | 6.873 | 5.727^efghi^ | 7.478 | 6.065 | 7.118 | 6.789^cde^ | 9.471 | 11.414 |
|  | May-W-0N | 5.523 | 4.856^efghij^ | 5.488 | 4.278^gh^ | 7.000 | 5.719^fghi^ | 7.473 | 6.007 | 7.031 | 6.759^cde^ | 9.544 | 11.593 |
|  | May-W-67N | 5.490 | 4.653^hij^ | 5.425 | 4.302^fgh^ | 7.107 | 5.370^ij^ | 7.761 | 6.063 | 7.224 | 6.586^def^ | 9.698 | 11.481 |
|  | Oct-NC-0N | 5.080 | 4.814^efghij^ | 5.448 | 4.302^fgh^ | 6.965 | 5.654^ghij^ | 7.498 | 6.381 | 7.432 | 6.789^cde^ | 9.578 | 11.652 |
|  | Oct-NC-67N | 5.115 | 5.095^def^ | 5.200 | 4.733^bcdefg^ | 6.975 | 6.042^cdefg^ | 7.576 | 6.595 | 7.456 | 7.317^a^ | 9.333 | 11.551 |
|  | Oct-V-0N | 5.232 | 5.122^de^ | 5.898 | 4.962^abcd^ | 7.055 | 6.139^bcd^ | 7.608 | 6.818 | 7.366 | 7.247^a^ | 9.496 | 11.662 |
|  | Oct-V-67N | 5.224 | 5.166^cde^ | 6.083 | 5.097^abc^ | 7.004 | 6.099^cdef^ | 7.587 | 6.852 | 7.476 | 7.170^ab^ | 9.335 | 11.855 |
|  | Oct-W-0N | 5.421 | 5.040^defgh^ | 5.568 | 4.627^defg^ | 6.923 | 5.574^hij^ | 7.487 | 6.182 | 7.222 | 7.035^abc^ | 9.379 | 11.683 |
|  | Oct-W-67N | 5.245 | 4.482^jk^ | 5.358 | 3.576^j^ | 6.936 | 5.270^j^ | 7.608 | 6.125 | 7.290 | 6.560^defg^ | 9.268 | 11.645 |
|  | Nov-NC-0N | 5.543 | 5.607^ab^ | 4.804 | 4.714^bcdefg^ | 7.054 | 6.654^a^ | 7.670 | 7.371 | 7.012 | 6.532^defghi^ | 9.517 | 11.451 |
|  | Nov-NC-67N | 5.499 | 5.525^abc^ | 4.822 | 5.241^a^ | 7.071 | 6.546^a^ | 7.822 | 7.476 | 7.135 | 6.788^cde^ | 9.432 | 11.509 |
|  | Nov-V-0N | 5.626 | 5.714^a^ | 5.294 | 5.105^ab^ | 7.152 | 6.299^abc^ | 7.796 | 7.156 | 7.229 | 6.541^defgh^ | 9.530 | 11.613 |
|  | Nov-V-67N | 5.441 | 5.821^a^ | 5.264 | 4.787^abcde^ | 7.075 | 6.520^ab^ | 7.763 | 7.177 | 7.219 | 6.648^def^ | 9.432 | 11.704 |
|  | Nov-W-0N | 5.436 | 5.316^bcd^ | 4.916 | 4.387^efgh^ | 7.078 | 5.857^defgh^ | 7.578 | 6.738 | 6.974 | 5.802^k^ | 9.346 | 11.478 |
|  | Nov-W-67N | 5.482 | 5.663^ab^ | 4.548 | 4.075^hi^ | 7.110 | 6.353^abc^ | 7.832 | 7.386 | 7.162 | 6.353^fghij^ | 9.434 | 11.695 |
| Season  *  Tillage  *  Cover  *  Nitrogen | Apr-NT-NC-0N | 5.580 | 3.671 | 4.602 | 3.109 | 6.951 | 5.148 | 7.323 | 5.252 | 6.848 | 5.533 | 9.318 | 10.487 |
|  | Apr-NT-NC-67N | 5.018 | 4.432 | 4.633 | 4.004 | 6.687 | 5.690 | 7.231 | 5.881 | 6.764 | 6.201 | 9.109 | 10.749 |
|  | Apr-NT-V-0N | 5.326 | 4.659 | 5.575 | 4.606 | 7.103 | 6.020 | 7.563 | 7.656 | 7.213 | 6.629 | 9.467 | 11.546 |
|  | Apr-NT-V-67N | 5.317 | 4.806 | 5.527 | 4.559 | 6.686 | 6.195 | 7.138 | 6.320 | 6.814 | 6.605 | 9.121 | 11.558 |
|  | Apr-NT-W-0N | 5.417 | 4.903 | 5.425 | 3.614 | 6.819 | 5.472 | 7.258 | 5.619 | 6.862 | 6.167 | 9.356 | 11.541 |
|  | Apr-NT-W-67N | 5.435 | 4.516 | 4.627 | 3.800 | 6.784 | 5.657 | 7.401 | 5.724 | 6.801 | 5.859 | 9.175 | 11.117 |
|  | Apr-CT-NC-0N | 5.678 | 4.631 | 5.129 | 4.105 | 7.043 | 6.095 | 7.515 | 6.119 | 7.007 | 6.488 | 9.395 | 11.290 |
|  | Apr-CT-NC-67N | 5.474 | 4.601 | 4.984 | 3.905 | 6.965 | 5.921 | 7.607 | 6.047 | 7.045 | 6.205 | 9.355 | 11.294 |
|  | Apr-CT-V-0N | 5.695 | 4.731 | 5.563 | 4.681 | 6.993 | 6.213 | 7.631 | 6.259 | 7.179 | 6.758 | 9.315 | 11.349 |
|  | Apr-CT-V-67N | 5.703 | 5.111 | 5.641 | 4.919 | 6.978 | 6.326 | 7.690 | 6.634 | 7.169 | 7.064 | 9.421 | 11.656 |
|  | Apr-CT-W-0N | 5.602 | 4.403 | 5.083 | 3.661 | 6.878 | 5.525 | 7.328 | 5.615 | 6.768 | 6.190 | 9.234 | 11.181 |
|  | Apr-CT-W-67N | 5.793 | 4.478 | 4.994 | 4.124 | 6.981 | 5.756 | 7.682 | 6.013 | 7.146 | 6.548 | 9.357 | 11.286 |
|  | May-NT-NC-0N | 5.350 | 4.009 | 4.975 | 3.682 | 6.901 | 5.119 | 7.301 | 5.352 | 6.881 | 6.085 | 9.497 | 11.508 |
|  | May-NT-NC-67N | 5.148 | 4.374 | 5.111 | 4.355 | 6.913 | 5.318 | 7.330 | 5.664 | 6.848 | 6.367 | 9.324 | 11.407 |
|  | May-NT-V-0N | 5.285 | 4.844 | 5.557 | 5.041 | 6.918 | 5.964 | 7.573 | 6.291 | 7.005 | 7.135 | 9.477 | 11.545 |
|  | May-NT-V-67N | 5.203 | 4.573 | 5.654 | 4.846 | 6.768 | 5.630 | 7.452 | 5.991 | 7.033 | 6.641 | 9.412 | 11.134 |
|  | May-NT-W-0N | 5.447 | 4.740 | 5.454 | 4.270 | 6.950 | 5.823 | 7.365 | 6.018 | 6.910 | 6.692 | 9.498 | 11.546 |
|  | May-NT-W-67N | 5.468 | 4.397 | 5.353 | 4.220 | 7.120 | 5.430 | 7.672 | 5.993 | 7.159 | 6.421 | 9.697 | 11.380 |
|  | May-CT-NC-0N | 5.634 | 4.958 | 5.365 | 4.306 | 6.970 | 5.671 | 7.615 | 6.221 | 7.097 | 6.820 | 9.653 | 11.620 |
|  | May-CT-NC-67N | 5.574 | 4.867 | 5.407 | 4.280 | 6.986 | 5.741 | 7.524 | 6.261 | 7.067 | 6.701 | 9.607 | 11.533 |
|  | May-CT-V-0N | 5.773 | 5.269 | 5.971 | 5.195 | 7.099 | 6.140 | 7.820 | 6.525 | 7.331 | 7.344 | 9.659 | 11.731 |
|  | May-CT-V-67N | 5.590 | 4.863 | 5.854 | 4.991 | 6.979 | 5.824 | 7.504 | 6.138 | 7.204 | 6.936 | 9.530 | 11.694 |
|  | May-CT-W-0N | 5.600 | 4.972 | 5.522 | 4.287 | 7.049 | 5.616 | 7.580 | 5.995 | 7.151 | 6.827 | 9.591 | 11.639 |
|  | May-CT-W-67N | 5.511 | 4.909 | 5.497 | 4.384 | 7.094 | 5.310 | 7.851 | 6.133 | 7.289 | 6.750 | 9.699 | 11.583 |
|  | Oct-NT-NC-0N | 4.837 | 4.631 | 5.446 | 4.025 | 6.907 | 5.527 | 7.414 | 6.504 | 7.426 | 6.700 | 9.491 | 11.742 |
|  | Oct-NT-NC-67N | 4.870 | 5.004 | 5.036 | 4.333 | 6.994 | 6.064 | 7.469 | 6.528 | 7.444 | 7.281 | 9.398 | 11.512 |
|  | Oct-NT-V-0N | 5.052 | 5.047 | 5.687 | 5.015 | 7.064 | 6.041 | 7.522 | 6.700 | 7.365 | 7.199 | 9.460 | 11.922 |
|  | Oct-NT-V-67N | 5.235 | 4.771 | 6.052 | 4.880 | 6.961 | 5.980 | 7.472 | 6.601 | 7.409 | 6.780 | 9.264 | 11.688 |
|  | Oct-NT-W-0N | 5.358 | 4.984 | 5.696 | 4.541 | 6.864 | 5.540 | 7.438 | 5.950 | 7.186 | 6.742 | 9.372 | 11.604 |
|  | Oct-NT-W-67N | 5.072 | 4.710 | 5.517 | 4.065 | 6.952 | 5.821 | 7.497 | 6.496 | 7.230 | 6.774 | 9.258 | 11.727 |
|  | Oct-CT-NC-0N | 5.322 | 4.997 | 5.450 | 4.579 | 7.024 | 5.782 | 7.583 | 6.258 | 7.438 | 6.878 | 9.665 | 11.561 |
|  | Oct-CT-NC-67N | 5.360 | 5.187 | 5.364 | 5.134 | 6.957 | 6.020 | 7.683 | 6.662 | 7.467 | 7.354 | 9.267 | 11.589 |
|  | Oct-CT-V-0N | 5.412 | 5.197 | 6.109 | 4.909 | 7.045 | 6.238 | 7.695 | 6.937 | 7.367 | 7.295 | 9.532 | 11.403 |
|  | Oct-CT-V-67N | 5.213 | 5.560 | 6.113 | 5.315 | 7.048 | 6.217 | 7.703 | 7.103 | 7.543 | 7.559 | 9.405 | 12.022 |
|  | Oct-CT-W-0N | 5.485 | 5.096 | 5.441 | 4.713 | 6.982 | 5.608 | 7.537 | 6.414 | 7.258 | 7.329 | 9.387 | 11.761 |
|  | Oct-CT-W-67N | 5.417 | 4.253 | 5.199 | 3.087 | 6.920 | 4.720 | 7.720 | 5.753 | 7.349 | 6.347 | 9.277 | 11.562 |
|  | Nov-NT-NC-0N | 5.541 | 5.790 | 4.939 | 5.150 | 7.110 | 7.109 | 7.681 | 7.643 | 7.063 | 6.817 | 9.580 | 11.548 |
|  | Nov-NT-NC-67N | 5.435 | 5.605 | 4.895 | 5.265 | 7.010 | 6.745 | 7.705 | 7.513 | 7.080 | 6.736 | 9.392 | 11.327 |
|  | Nov-NT-V-0N | 5.448 | 5.725 | 5.327 | 5.243 | 7.116 | 6.406 | 7.689 | 7.189 | 7.144 | 6.622 | 9.499 | 11.694 |
|  | Nov-NT-V-67N | 5.424 | 6.009 | 5.335 | 5.000 | 7.046 | 6.573 | 7.751 | 7.148 | 7.233 | 6.842 | 9.289 | 11.770 |
|  | Nov-NT-W-0N | 5.342 | 4.994 | 4.805 | 4.242 | 6.959 | 5.205 | 7.537 | 6.197 | 6.891 | 5.493 | 9.161 | 11.481 |
|  | Nov-NT-W-67N | 5.391 | 5.523 | 4.511 | 4.064 | 7.051 | 6.185 | 7.756 | 7.207 | 7.094 | 6.349 | 9.284 | 11.586 |
|  | Nov-CT-NC-0N | 5.545 | 5.425 | 4.669 | 4.278 | 6.997 | 6.199 | 7.659 | 7.099 | 6.961 | 6.247 | 9.453 | 11.354 |
|  | Nov-CT-NC-67N | 5.564 | 5.445 | 4.750 | 5.216 | 7.132 | 6.348 | 7.938 | 7.439 | 7.190 | 6.840 | 9.472 | 11.691 |
|  | Nov-CT-V-0N | 5.805 | 5.702 | 5.261 | 4.967 | 7.188 | 6.192 | 7.903 | 7.124 | 7.314 | 6.459 | 9.561 | 11.532 |
|  | Nov-CT-V-67N | 5.459 | 5.632 | 5.192 | 4.575 | 7.104 | 6.467 | 7.775 | 7.205 | 7.206 | 6.454 | 9.574 | 11.638 |
|  | Nov-CT-W-0N | 5.530 | 5.639 | 5.027 | 4.532 | 7.198 | 6.508 | 7.618 | 7.279 | 7.057 | 6.111 | 9.531 | 11.475 |
|  | Nov-CT-W-67N | 5.573 | 5.803 | 4.584 | 4.087 | 7.168 | 6.521 | 7.907 | 7.566 | 7.229 | 6.357 | 9.584 | 11.803 |

^†^NC = no cover; V = vetch; W = wheat; NT = no tillage; CT = conventional tillage; 0N = no fertilization; 67N = 67 kg N ha^-1^ fertilization.

^‡^AOB = Ammonia oxidizing bacteria.
