# Supplementary material for: Soil Health Management Enhances Microbial Nitrogen Cycling Capacity and Activity
Source: mSphere. 2021 Jan 13;6(1):e01237-20. doi: 10.1128/mSphere.01237-20 (PMC7845608; doi:10.1128/mSphere.01237-20)
Supplement: TABLE S6 [file mSphere.01237-20_st006.docx]

| Treatment^†^ | | *nifH* | AOB^‡^ *amoA* | *nirK* | *nirS* | *nosZ* |
| --- | --- | --- | --- | --- | --- | --- |
| Season | April | -0.925^d^ | -1.080^b^ | -1.071^b^ | -1.411^c^ | -0.614^b^ |
|  | May | -0.734^c^ | -0.988^b^ | -1.347^c^ | -1.500^c^ | -0.355^a^ |
|  | October | -0.266^b^ | -1.043^b^ | -1.180^bc^ | -1.069^b^ | -0.354^a^ |
|  | November | 0.103^a^ | -0.223^a^ | -0.719^a^ | -0.526^a^ | -0.678^b^ |
| Cover | NC | -0.519^b^ | -0.706^a^ | -1.066^b^ | -1.133^b^ | -0.523^b^ |
|  | V | -0.277^a^ | -0.730^a^ | -0.854^a^ | -0.915^a^ | -0.325^a^ |
|  | W | -0.570^b^ | -1.065^b^ | -1.317^c^ | -1.323^c^ | -0.652^b^ |
| Season  *  Cover | Apr-NC | -1.104 | -1.123^efgh^ | -1.198^def^ | -1.594^ef^ | -0.809^ef^ |
|  | Apr-V | -0.683 | -0.885^def^ | -0.751^abc^ | -0.934^bc^ | -0.330^ab^ |
|  | Apr-W | -0.987 | -1.233^gh^ | -1.263^efg^ | -1.674^f^ | -0.703^de^ |
|  | May-NC | -0.875 | -1.059^defgh^ | -1.480^fg^ | -1.568^ef^ | -0.480^bcd^ |
|  | May-V | -0.576 | -0.740^cd^ | -1.051^cde^ | -1.351^de^ | -0.129^a^ |
|  | May-W | -0.752 | -1.166^fgh^ | -1.509^g^ | -1.582^ef^ | -0.455^bcd^ |
|  | Oct-NC | -0.143 | -0.806^cde^ | -1.122^de^ | -1.049^cd^ | -0.391^abc^ |
|  | Oct-V | -0.084 | -0.961^defg^ | -0.911^bcd^ | -0.763^bc^ | -0.213^ab^ |
|  | Oct-W | -0.572 | -1.362^h^ | -1.508^g^ | -1.395^ef^ | -0.458^bcd^ |
|  | Nov-NC | 0.045 | 0.164^a^ | -0.462^a^ | -0.322^a^ | -0.413^bc^ |
|  | Nov-V | 0.233 | -0.333^b^ | -0.704^ab^ | -0.613^ab^ | -0.630^cde^ |
|  | Nov-W | 0.031 | -0.501^bc^ | -0.989^bcde^ | -0.643^b^ | -0.990^f^ |
| Season  *  Nitrogen | Apr-0N | -1.050 | -1.267 | -1.219^de^ | -1.469 | -0.685^cd^ |
|  | Apr-67N | -0.799 | -0.894 | -0.923^bc^ | -1.355 | -0.543^bc^ |
|  | May-0N | -0.716 | -1.010 | -1.259^de^ | -1.475 | -0.246^a^ |
|  | May-67N | -0.752 | -0.967 | -1.434^e^ | -1.525 | -0.464^abc^ |
|  | Oct-0N | -0.252 | -1.008 | -1.192^de^ | -1.071 | -0.316^a^ |
|  | Oct-67N | -0.280 | -1.078 | -1.168^cd^ | -1.067 | -0.391^ab^ |
|  | Nov-0N | 0.011 | -0.270 | -0.825^ab^ | -0.593 | -0.780^d^ |
|  | Nov-67N | 0.195 | -0.177 | -0.612^a^ | -0.459 | -0.576^bcd^ |
| Cover  *  Nitrogen | NC-0N | -0.672^c^ | -0.918^bc^ | -1.157 | -1.205 | -0.644^c^ |
|  | NC-67N | -0.366^ab^ | -0.494^a^ | -0.975 | -1.061 | -0.403^ab^ |
|  | V-0N | -0.328^ab^ | -0.674^ab^ | -0.914 | -0.911 | -0.310^a^ |
|  | V-67N | -0.227^a^ | -0.786^b^ | -0.795 | -0.918 | -0.341^a^ |
|  | W-0N | -0.506^bc^ | -1.074^c^ | -1.300 | -1.322 | -0.567^bc^ |
|  | W-67N | -0.634^c^ | -1.057^c^ | -1.334 | -1.325 | -0.737^c^ |
| Season  *  Cover  *  Tillage | Apr-NC-NT | -1.247^k^ | -1.194 | -1.400^fghi^ | -1.710^ij^ | -0.939 |
|  | Apr-NC-CT | -0.960^ijk^ | -1.051 | -0.996^bcdef^ | -1.478^fghij^ | -0.680 |
|  | Apr-V-NT | -0.589^fghi^ | -0.969 | -0.787^bcd^ | -0.614^bc^ | -0.396 |
|  | Apr-V-CT | -0.778^hij^ | -0.802 | -0.716^bc^ | -1.214^defg^ | -0.263 |
|  | Apr-W-NT | -0.717^hij^ | -1.319 | -1.237^efg^ | -1.658^ghij^ | -0.819 |
|  | Apr-W-CT | -1.257^k^ | -1.147 | -1.289^fgh^ | -1.691^hij^ | -0.588 |
|  | May-NC-NT | -1.057^jk^ | -1.024 | -1.688^hi^ | -1.808^j^ | -0.639 |
|  | May-NC-CT | -0.692^ghij^ | -1.093 | -1.272^fgh^ | -1.329^efghi^ | -0.322 |
|  | May-V-NT | -0.536^efghi^ | -0.662 | -1.046^cdef^ | -1.371^efghij^ | -0.131 |
|  | May-V-CT | -0.615^fghij^ | -0.819 | -1.057^cdef^ | -1.330^efghi^ | -0.128 |
|  | May-W-NT | -0.889^ijk^ | -1.158 | -1.409^fghi^ | -1.513^fghij^ | -0.478 |
|  | May-W-CT | -0.615^fghij^ | -1.174 | -1.608^ghi^ | -1.651^ghij^ | -0.432 |
|  | Oct-NC-NT | -0.036^bcd^ | -1.062 | -1.155^def^ | -0.925^cde^ | -0.445 |
|  | Oct-NC-CT | -0.249^cdefg^ | -0.550 | -1.090^cdef^ | -1.172^def^ | -0.337 |
|  | Oct-V-NT | -0.234^bcdef^ | -0.922 | -1.002^bcdef^ | -0.846^cd^ | -0.397 |
|  | Oct-V-CT | -0.124^abcd^ | -0.999 | -0.819^bcde^ | -0.679^bc^ | -0.028 |
|  | Oct-W-NT | -0.367^defgh^ | -1.303 | -1.228^efg^ | -1.244^defgh^ | -0.450 |
|  | Oct-W-CT | -0.777^hij^ | -1.420 | -1.788^i^ | -1.545^fghij^ | -0.466 |
|  | Nov-NC-NT | 0.210^ab^ | 0.290 | -0.133^a^ | -0.115^a^ | -0.295 |
|  | Nov-NC-CT | -0.119^bcde^ | 0.037 | -0.791^bcd^ | -0.529^abc^ | -0.532 |
|  | Nov-V-NT | 0.347^a^ | -0.210 | -0.591^b^ | -0.551^abc^ | -0.456 |
|  | Nov-V-CT | 0.035^abcd^ | -0.456 | -0.817^bcde^ | -0.674^bc^ | -0.804 |
|  | Nov-W-NT | -0.108^bcde^ | -0.505 | -1.310^fgh^ | -0.945^cde^ | -1.072 |
|  | Nov-W-CT | 0.030^abc^ | -0.496 | -0.668^bc^ | -0.340^ab^ | -0.909 |
| Season  *  Cover  *  Nitrogen | Apr-NC-0N | -1.478 | -1.259^gh^ | -1.376 | -1.733 | -0.917^jk^ |
|  | Apr-NC-67N | -0.729 | -0.987^efg^ | -1.020 | -1.455 | -0.702^ghij^ |
|  | Apr-V-0N | -0.815 | -0.925^defg^ | -0.932 | -0.930 | -0.502^bcdefghi^ |
|  | Apr-V-67N | -0.551 | -0.845^defg^ | -0.571 | -0.937 | -0.157^abc^ |
|  | Apr-W-0N | -0.856 | -1.616^hi^ | -1.350 | -1.676 | -0.637^efghij^ |
|  | Apr-W-67N | -1.118 | -0.849^defg^ | -1.176 | -1.673 | -0.770^ij^ |
|  | May-NC-0N | -1.008 | -1.176^gh^ | -1.541 | -1.672 | -0.537^cdefghij^ |
|  | May-NC-67N | -0.741 | -0.941^defg^ | -1.420 | -1.465 | -0.424^bcdefghi^ |
|  | May-V-0N | -0.472 | -0.646^cdef^ | -0.957 | -1.288 | 0.071^a^ |
|  | May-V-67N | -0.679 | -0.835^defg^ | -1.146 | -1.413 | -0.330^bcdefg^ |
|  | May-W-0N | -0.667 | -1.209^gh^ | -1.280 | -1.466 | -0.271^abcde^ |
|  | May-W-67N | -0.837 | -1.123^fg^ | -1.737 | -1.699 | -0.638^efghij^ |
|  | Oct-NC-0N | -0.266 | -1.145^gh^ | -1.311 | -1.117 | -0.643^efghij^ |
|  | Oct-NC-67N | -0.020 | -0.467^bcd^ | -0.933 | -0.981 | -0.139^ab^ |
|  | Oct-V-0N | -0.110 | -0.936^defg^ | -0.915 | -0.790 | -0.119^ab^ |
|  | Oct-V-67N | -0.265 | -0.985^efg^ | -0.906 | -0.735 | -0.306^abcdef^ |
|  | Oct-W-0N | -0.381 | -0.941^defg^ | -1.349 | -1.305 | -0.186^abcd^ |
|  | Oct-W-67N | -0.763 | -1.782^i^ | -1.666 | -1.484 | -0.729^hij^ |
|  | Nov-NC-0N | 0.064 | -0.091^b^ | -0.400 | -0.299 | -0.480^bcdefghi^ |
|  | Nov-NC-67N | 0.026 | 0.418^a^ | -0.525 | -0.346 | -0.347^bcdefgh^ |
|  | Nov-V-0N | 0.087 | -0.189^bc^ | -0.853 | -0.640 | -0.688^fghij^ |
|  | Nov-V-67N | 0.288 | -0.476^bcd^ | -0.555 | -0.586 | -0.572^defghij^ |
|  | Nov-W-0N | -0.300 | -0.529^bcde^ | -1.222 | -0.840 | -1.172^k^ |
|  | Nov-W-67N | 0.181 | -0.472^bcd^ | -0.757 | -0.445 | -0.809^ijk^ |

^†^NC = no cover; V = vetch; W = wheat; NT = no tillage; CT = conventional tillage; 0N = no fertilization; 67N = 67 kg N ha^-1^ fertilization.

^‡^AOB = Ammonia oxidizing bacteria.
