# Supplementary material for: Soil Health Management Enhances Microbial Nitrogen Cycling Capacity and Activity
Source: mSphere. 2021 Jan 13;6(1):e01237-20. doi: 10.1128/mSphere.01237-20 (PMC7845608; doi:10.1128/mSphere.01237-20)
Supplement: TABLE S7 [file mSphere.01237-20_st007.docx]

| Sample type | Treatment | Group | | R | *p*-value |
| --- | --- | --- | --- | --- | --- |
| Soil DNA  (gene level) | Season | Overall level | | 0.1748 | 0.001^***^ |
|  |  | Within group | April/May | 0.1039 | 0.001^***^ |
|  |  |  | April/October | 0.1194 | 0.001^***^ |
|  |  |  | April/November | 0.0495 | 0.011^*^ |
|  |  |  | May/October | 0.3101 | 0.001^***^ |
|  |  |  | May/November | 0.2751 | 0.001^***^ |
|  |  |  | October/November | 0.1764 | 0.001^***^ |
|  | Winter cover crop | Overall level | | 0.0150 | 0.060 |
|  |  | Within group | NC/V^†^ | 0.0361 | 0.017^*^ |
|  |  |  | NC/W | 0.0007 | 0.362 |
|  |  |  | V/W | 0.0085 | 0.185 |
|  | Tillage | NT/CT | | 0.0222 | 0.020^*^ |
|  | Fertilization | 0N/67N | | 0.0401 | 0.003^**^ |
| Soil RNA  (transcription level) | Season | Overall level | | 0.2656 | 0.001^***^ |
|  |  | Within group | April/May | 0.0597 | 0.004^**^ |
|  |  |  | April/October | 0.0610 | 0.001^***^ |
|  |  |  | April/November | 0.3793 | 0.001^***^ |
|  |  |  | May/October | 0.0204 | 0.087 |
|  |  |  | May/November | 0.5814 | 0.001^***^ |
|  |  |  | October/November | 0.4872 | 0.001^***^ |
|  | Winter cover crop | Overall level | | 0.0495 | 0.001^***^ |
|  |  | Within group | NC/V | 0.0335 | 0.009^**^ |
|  |  |  | NC/W | 0.0254 | 0.029^*^ |
|  |  |  | V/W | 0.0953 | 0.001^***^ |
|  | Tillage | NT/CT | | 0.0205 | 0.016^*^ |
|  | Fertilization | 0N/67N | | -0.0053 | 0.819 |

Note: * *p*-value ≤ 0.05, ** *p*-value ≤ 0.01, *** *p*-value ≤ 0.001.

^†^NC = no cover; V = vetch; W = wheat; NT = no tillage; CT = conventional tillage; 0N = no fertilization; 67N = 67 kg N ha^-1^ fertilization.
